# Human **β**-defensin-3 attenuates atopic dermatitis–like inflammation through autophagy activation and the aryl hydrocarbon receptor signaling pathway

**DOI:** 10.1172/JCI156501

**Published:** 2022-09-01

**Authors:** Ge Peng, Saya Tsukamoto, Risa Ikutama, Hai Le Thanh Nguyen, Yoshie Umehara, Juan V. Trujillo-Paez, Hainan Yue, Miho Takahashi, Takasuke Ogawa, Ryoma Kishi, Mitsutoshi Tominaga, Kenji Takamori, Jiro Kitaura, Shun Kageyama, Masaaki Komatsu, Ko Okumura, Hideoki Ogawa, Shigaku Ikeda, François Niyonsaba

**Affiliations:** 1 Atopy (Allergy) Research Center and; 2 Department of Dermatology and Allergology, Juntendo University Graduate School of Medicine, Tokyo, Japan.; 3 Juntendo Itch Research Center, Institute for Environmental and Gender-Specific Medicine, Juntendo University Graduate School of Medicine, Urayasu, Japan.; 4 Department of Dermatology, Juntendo University Urayasu Hospital, Urayasu, Japan.; 5 Department of Physiology, Juntendo University Graduate School of Medicine, Tokyo, Japan.; 6 Faculty of International Liberal Arts, Juntendo University, Tokyo, Japan.

**Keywords:** Dermatology, Inflammation, Autophagy, Defensins, Tight junctions

## Abstract

Human β-defensin-3 (hBD-3) exhibits antimicrobial and immunomodulatory activities; however, its contribution to autophagy regulation remains unclear, and the role of autophagy in the regulation of the epidermal barrier in atopic dermatitis (AD) is poorly understood. Here, keratinocyte autophagy was restrained in the skin lesions of patients with AD and murine models of AD. Interestingly, hBD-3 alleviated the IL-4– and IL-13–mediated impairment of the tight junction (TJ) barrier through keratinocyte autophagy activation, which involved aryl hydrocarbon receptor (AhR) signaling. While autophagy deficiency impaired the epidermal barrier and exacerbated inflammation, hBD-3 attenuated skin inflammation and enhanced the TJ barrier in AD. Importantly, hBD-3–mediated improvement of the TJ barrier was abolished in autophagy-deficient AD mice and in AhR-suppressed AD mice, suggesting a role for hBD-3–mediated autophagy in the regulation of the epidermal barrier and inflammation in AD. Thus, autophagy contributes to the pathogenesis of AD, and hBD-3 could be used for therapeutic purposes.

## Graphical abstract

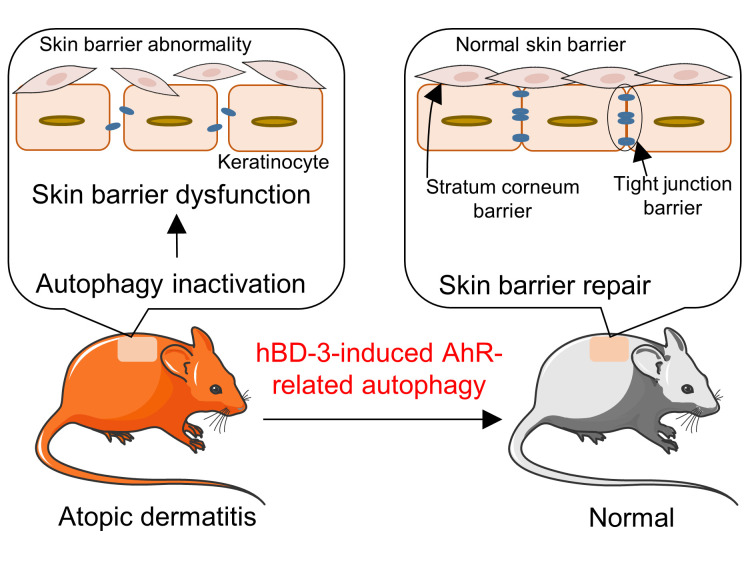

## Introduction

Atopic dermatitis (AD) is the most common inflammatory skin disorder, and it has a complex etiology that is dependent on interactions between the skin barrier and the environment (1). Emerging evidence indicates that the skin barrier has important roles in immune surveillance and homeostasis (2). In particular, inherited defects in epidermal barrier proteins facilitate the interaction between external antigens and skin-resident immune cells, resulting in local inflammation (1, 3). Inflammation may in turn cause skin barrier damage, which further exacerbates inflammation and allergic sensitization to environmental allergens (4). These observations suggest that it is important to maintain skin barrier function for both the effective management of AD and the prevention of the subsequent development of allergic diseases.

Autophagy is an essential process through which the cell breaks down unwanted components to maintain homeostasis, and it is typically triggered by nutrient starvation (5). Upregulation of microtubule-associated protein light chain 3-II (LC3-II) and downregulation of sequestosome-1/p62 (p62) result in autophagy activation (5). p62 acts as a selective autophagy adaptor and enhances inflammation in skin conditions, including AD and psoriasis, which are characterized by defects in the epidermal barrier and keratinocyte differentiation, through signaling of nuclear factor κ light chain enhancer of activated B cells (NF-κB) and mechanistic target of rapamycin (mTOR) (6). mTOR functions as an upstream regulator of autophagy. Activation of mTOR suppresses autophagy, while its inhibition initiates autophagy (7, 8), and it is associated with a defective epidermal barrier (9). Interestingly, fasting and dietary restriction, which activate autophagy, have been shown to improve the symptoms of allergic dermatitis (10), implying that there are system-wide benefits of autophagy activation. In addition, the application of rapamycin, an autophagy inducer, ameliorates AD-like skin lesions in NC/Nga mice (11). Notably, an association between AD and autophagy-related (ATG) genes, such as *ATG16L2*, *ATG4s*, and unc-51–like autophagy activating kinase (*ULK1*), has also been proposed (12). Furthermore, given that autophagosome-lysosome fusion supports epidermal differentiation (13) and that functional lysosome-related proteins such as cathepsins D and L are downregulated in AD skin lesions (14), it appears that autophagy plays a crucial role in AD.

Recent studies have demonstrated that autophagy contributes to keratinocyte differentiation, host defense, and immune responses in the epidermal barrier (15). The multilayered structure of the epidermis is continuously renewed by basal-layer keratinocytes that differentiate to form a physical barrier that is mainly composed of the stratum corneum (SC) barrier and tight junction (TJ) barrier. Autophagy contributes to the mature differentiation and development of the epidermis via the regulation of recycling endosomes (16), and ablation of autophagy suppresses the expression of differentiation markers in keratinocytes (17) and the epidermis (18). In addition, *Staphylococcus aureus*, which frequently colonizes AD skin, can persist within keratinocytes by exploiting autophagy (19). Activation of autophagy attenuates Toll-like receptor 3–mediated (TLR3-mediated) inflammatory responses in epidermal keratinocytes (20), while higher TLR3 expression is correlated with the occurrence of severe lesions of AD (21). These findings indicate that degradative autophagy is involved in the physiological mechanisms of the epidermal barrier.

In addition to antimicrobial activities, human β-defensin-3 (hBD-3) participates in pleiotropic immunomodulatory processes, including keratinocyte cytokine/chemokine production, cell proliferation, migration and differentiation, and the regulation of skin barrier function (22). hBD-3 is also involved in the pathogenesis of various skin diseases, including AD, in which abnormal expression of autophagy-related proteins was recently reported (12, 22, 23); however, the precise role of hBD-3 in autophagy regulation remains unclear, and the contribution of autophagy to epidermal barrier function in AD is also poorly understood.

Although nutrient starvation–induced autophagy enhances TJ barrier function in intestinal epithelial cells (24), little information is currently available on the association of autophagy with the skin barrier in AD. Here, we reveal an immunoregulatory mechanism of autophagy in AD and highlight the therapeutic role of the skin-derived antimicrobial peptide hBD-3 in AD, whose effects are mediated through the regulation of autophagy.

## Results

### Autophagy-related proteins are functionally inactive in AD skin lesions.

**Figure 1:**
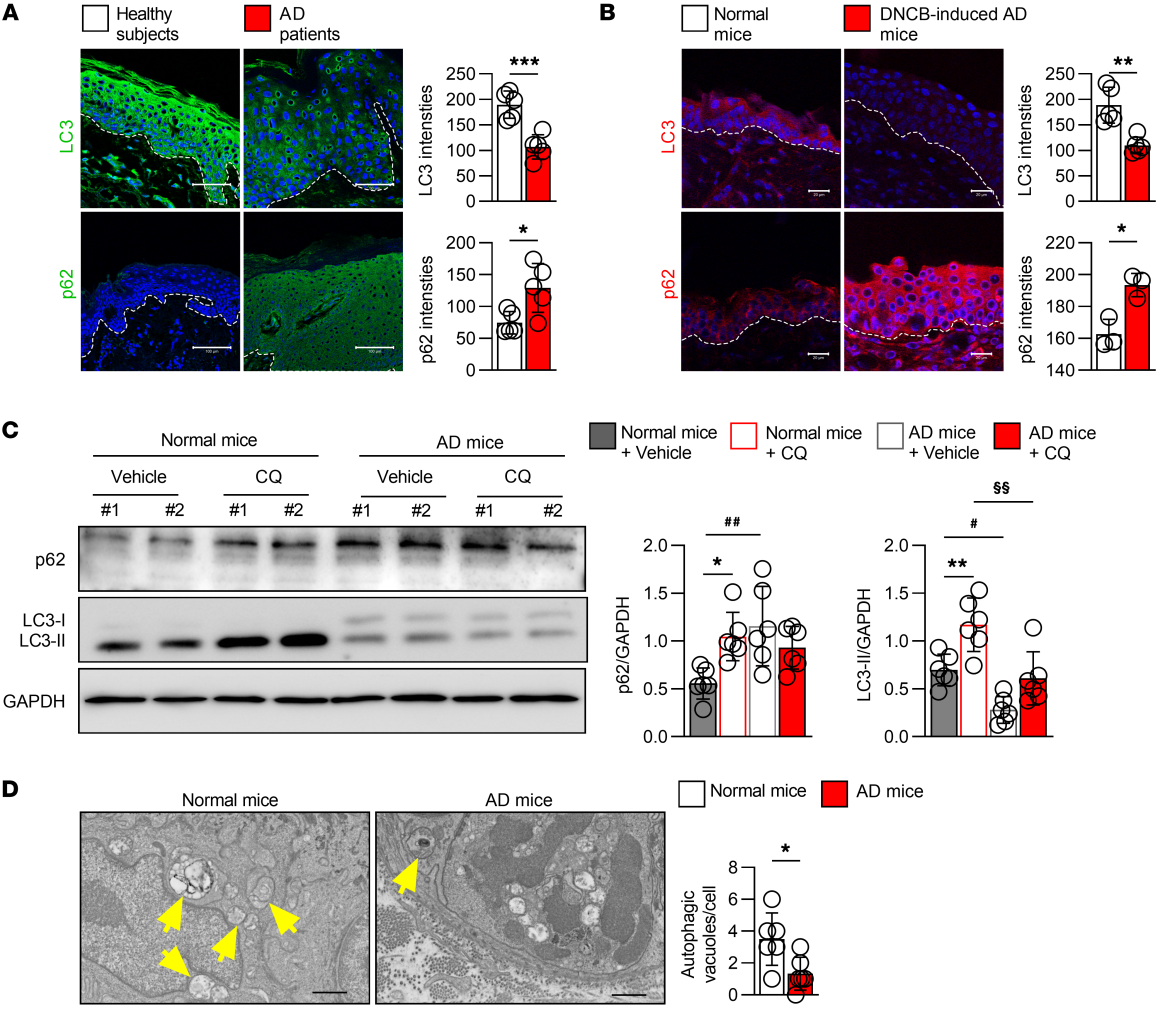
Autophagy-related proteins are dysregulated in AD skin lesions. (**A**) Immunofluorescence staining of LC3 and p62 in the epidermis of patients with AD and normal participants. Representative immunofluorescence images of skin (left) and quantification of the staining intensity in the epidermis (right). The white dashed line indicates the basement membrane between the epidermis and dermis. Scale bars: 50 or 100 μm; *n* = 5 per group. (**B**) Immunofluorescence staining of LC3 and p62 in the epidermis of DNCB-treated AD mice and normal mice. Representative immunofluorescence images of skin (left) and quantification of the staining intensity in the epidermis (right). The white dashed line indicates the basement membrane between the epidermis and dermis. Scale bars: 20 μm; *n* = 3–6 per group. (**C**) Expression of p62 and LC3 in the back skins of DNCB-induced AD mice and normal mice; *n* = 6 per group. Representative immunoblots of the indicated proteins from mouse skin lysates (left) and quantification of the band intensities of LC3 and p62 (right). GAPDH was used as a loading control. (**D**) Representative electron microscopic images of keratinocytes in lesional skin from DNCB-induced AD mice and keratinocytes in normal mouse skin (left) and quantification of autophagic vacuoles (right). The yellow arrowheads indicate autophagic vacuoles. Scale bars: 5 μm. Mean ± SD. *P < 0.05, **P < 0.01, ***P < 0.001, ^#^*P* < 0.05, ^##^*P* < 0.01, ^§§^*P* < 0.01. Statistical significance was determined by 2-tailed Student’s *t* test. All of the data are representative of 3 independent experiments.

LC3, which comprises cytosolic LC3-I and lipidated LC3-II, and p62 are widely used autophagy markers in mammalian cells (5). Following activation of autophagy, LC3-I is converted to LC3-II, leading to higher expression of LC3-II. At the same time, p62 is degraded, resulting in lower expression. To determine the autophagic status in AD, the expression patterns of LC3 and p62 were analyzed in skin biopsies from patients with AD and compared with those from normal healthy volunteers. In the epidermis of healthy volunteers, LC3 was displayed in all epidermal layers, with the strongest expression in the upper layer, which is consistent with a previous report (25), while this expression was remarkably reduced in the skin lesions of patients with AD (Figure 1A, top panels). In contrast, while p62 was absent in the epidermal layers of healthy skin, it accumulated in the parakeratotic regions of the AD epidermis (Figure 1A, bottom panels).

Furthermore, microarray data of skin lesions from 84 patients with AD and normal skin from 199 healthy volunteers were obtained from the ArrayExpress database (Supplemental Figure 1A; supplemental material available online with this article; https://doi.org/10.1172/JCI156501DS1). A comparison of the differences in the ATG genes between the 2 groups revealed that the fold changes in the gene expression of *LC3B* and *ULK1*, as well as mitophagy-related genes such as *PINK1* and *PARK2*, were significantly downregulated in patients with AD (Supplemental Figure 1, B and C). This observation implies that autophagy dysregulation in skin might be involved in AD pathogenesis.

To confirm whether autophagy is dysregulated in AD, a 2,4-dinitrochlorobenzene–induced (DNCB-induced) AD-like mouse model (26) was established. Similar to the results seen in patients with AD, LC3 was downregulated, while p62 was increased in the epidermis of AD mice compared with normal mice (Figure 1B). We further established 2 other AD murine models, an AD-like mouse model induced by the vitamin D_3_ analog MC903 (27) and a *Dermatophagoides farinae* extract–induced NC/Nga AD model (28). Consistently, decreases in LC3 and increases in p62 in skin samples from both AD models were observed (Supplemental Figure 1, D and E), further confirming that autophagy is inactivated in AD skin.

The difference in the levels of LC3-II between samples in the absence and presence of lysosome inhibitors represents the level of autophagic flux (5). To measure autophagic flux in mouse skin, we injected the lysosome inhibitor chloroquine (CQ) subcutaneously 4 hours before skin tissue collection. Immunoblot analysis showed that LC3-II levels were markedly decreased in the skin tissues from AD mice compared with those from normal mice in the absence of CQ treatment. Likewise, CQ treatment increased the levels of LC3-II and p62 in skin tissues from normal mice, while there was no remarkable difference among AD mice, indicating partial blockade of autophagic flux in the skin tissues of AD mice (Figure 1C). Moreover, ultrastructural transmission electron microscopy image analysis revealed that keratinocytes of DNCB-induced AD skin lesions exhibited fewer autophagic vesicles than those in normal mouse skin (Figure 1D), suggesting that autophagy activation in keratinocytes might play a crucial role in AD pathogenesis.

### Th2-derived cytokines are involved in autophagy inactivation in AD keratinocytes.

**Figure 2:**
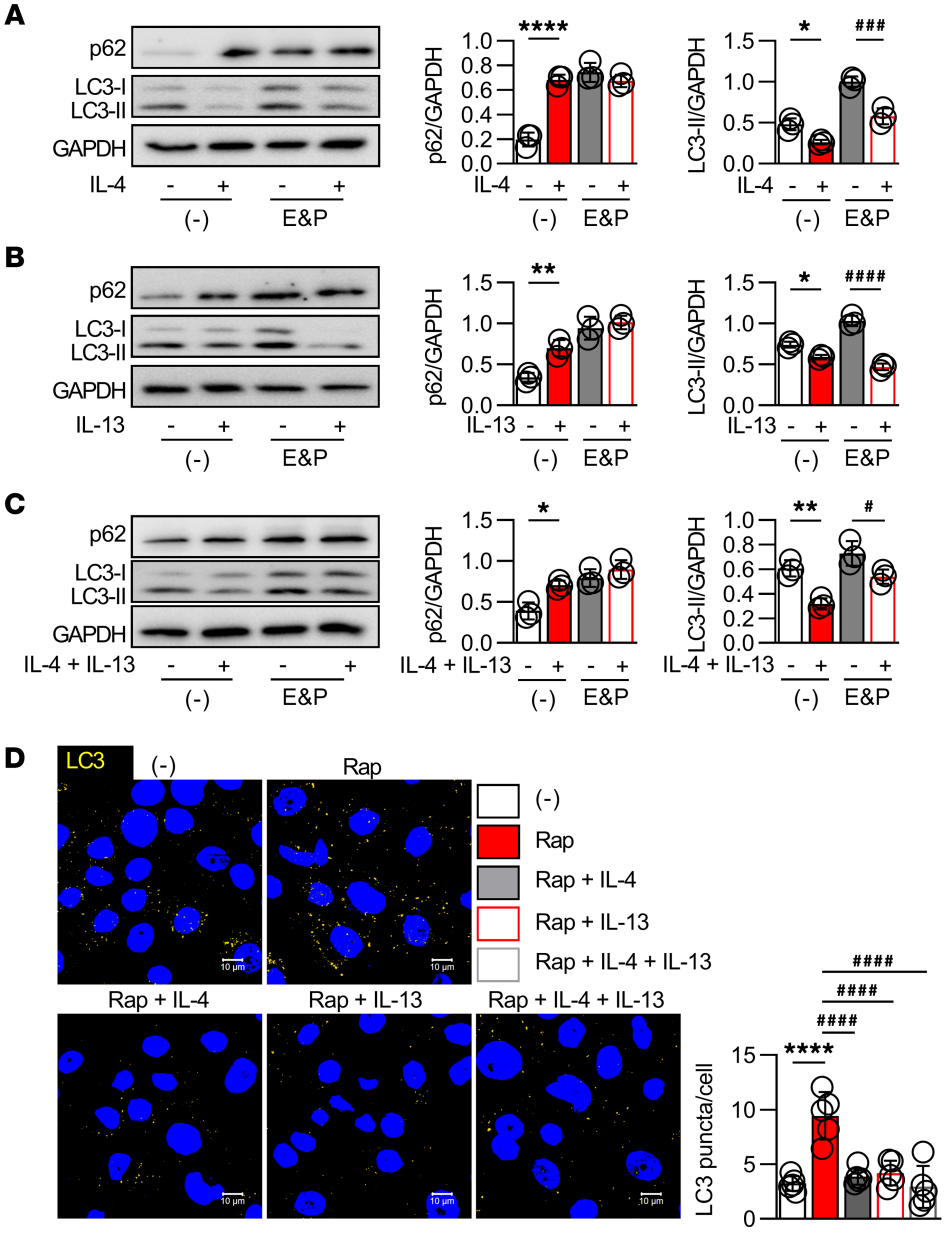
Th2-derived cytokines are involved in the inactivation of autophagy in AD keratinocytes. (**A**–**C**) Keratinocytes were stimulated for 12 hours with 100 ng/mL IL-4 or IL-13 alone or in combination in the presence (+) or absence (–) of 10 μg/mL E&P; *n* = 3 per group. Representative p62 and LC3 immunoblots (left) and quantification of band intensities (right). GAPDH was used as a loading control. (**D**) Keratinocytes were stimulated for 12 hours with or without IL-4 or IL-13 alone or in combination in the presence of 10 μM rapamycin (Rap); *n* = 5 per group. Representative immunofluorescence images (left) and quantification of LC3 puncta in keratinocytes (right). Scale bars: 10 μm. Mean ± SD. *P < 0.05, **P < 0.01, ****P < 0.0001, ^#^*P* < 0.05, ^###^*P* < 0.001, ^####^*P* < 0.0001. Statistical significance was determined by 2-tailed Student’s *t* test or 1-way ANOVA with Tukey’s multiple-comparison test. All of the data are representative of 3 independent experiments.

T helper type 2 (Th2) cytokines, such as interleukin-4 (IL-4) and IL-13, have long been associated with the pathogenesis of AD (1), and keratinocytes treated with a mixture of IL-4 and IL-13 constitute an in vitro AD-like keratinocyte model (29). The effects of IL-4 and IL-13 on human keratinocyte autophagy were evaluated in the presence or absence of the lysosomal enzyme inhibitors E64d and pepstatin A (E&P), which prevent lysosomal acidification and autophagosome-lysosome fusion and are used to exclude the possibility of simple blockade of lysosomal degradation rather than autophagy activation (30). As shown in Figure 2, treatment of keratinocytes with IL-4 or IL-13 alone and the combination of both IL-4 and IL-13 significantly increased p62 levels in the absence of E&P, while there was no difference in p62 levels in the presence of E&P. These cytokines markedly reduced the LC3-II amounts in the presence of E&P, indicating that IL-4 and IL-13 partially block autophagic flux in keratinocytes (Figure 2, A–C), which is consistent with the in vivo results described in Figure 1C.

Moreover, while the autophagy inducer rapamycin increased the appearance of LC3-positive puncta, an indicator of autophagy occurrence (5), the administration of IL-4 or IL-13 alone as well as their combination significantly diminished the number of LC3-positive puncta in rapamycin-treated keratinocytes (Figure 2D). Interestingly, treatment of keratinocytes with other Th2 cytokines, such as IL-33 and thymic stromal lymphopoietin (TSLP), affected neither p62 accumulation nor LC3-II levels in the presence of E&P (Supplemental Figure 2, A and B) and did not affect the number of LC3-positive puncta in keratinocytes (Supplemental Figure 2F), implying that not all Th2 cytokines involved in AD pathogenesis play a role in the autophagy process in keratinocytes.

Given that the activation of Th1- and Th17-mediated responses has been reported in chronic AD skin lesions (1), we investigated the effects of Th1 and Th17 cytokines on keratinocyte autophagy. Treatment with the Th1 cytokine interferon-γ (IFN-γ) and the Th17 cytokine IL-17 decreased the accumulation of p62 and increased the LC3-II amounts in keratinocytes, while IL-23 did not show any significant effect (Supplemental Figure 2, C–E). Interestingly, both IFN-γ and IL-17 significantly increased LC3-II levels in the presence of E&P compared with the absence of E&P (Supplemental Figure 2, C and D), suggesting that these cytokines may induce activation rather than inhibition of autophagic flux in keratinocytes. Taken together, the Th2 cytokines IL-4 and IL-13 may inhibit autophagic flux in keratinocytes.

### Keratinocyte-specific autophagy deficiency exacerbates AD.

Although autophagy is involved in the homeostasis of intestinal barrier function (24), the role of autophagy in epidermal barrier function remains unclear. To examine the role of autophagy in the maintenance of the barrier function in keratinocytes, we established autophagy-deficient keratinocytes by transfecting mutant Atg3C264S, which has a mutation at the active-site cysteine that leads to autophagy inactivation, using an adenovirus system as described in a previous study (31). Note that Atg3 is an E2-like enzyme required for Atg8 conjugation, which is indispensable for the proper development of autophagic isolation membranes (31). Following Atg3C264S transfection into keratinocytes, Atg3 expression was not affected, while LC3-II was decreased and p62 was increased in the transfected cells, confirming the deficiency of autophagy in these cells and the equal transfection levels between the Atg3- and Atg3C264S-transduced cells (Supplemental Figure 3A). In autophagy-deficient keratinocytes, the mRNA expression of TJ-related proteins, including claudin-1 and zonula occludens-1 (ZO-1; tight junction protein 1 [TJP1]), and the expression of SC barrier proteins, such as filaggrin and loricrin, were significantly decreased (Supplemental Figure 3B). Likewise, claudin-1 and ZO-1 were noticeably reduced at the protein level, and a similar tendency was observed for filaggrin and loricrin expression, although it was not significant (Supplemental Figure 3C). We further confirmed that the intercellular distribution of claudin-1 and ZO-1 was attenuated in autophagy-deficient keratinocytes (Supplemental Figure 3D). Both claudin-1 and ZO-1 are important TJ barrier components, as claudin-1–null mice die within 1 day of birth owing to dehydration (32), and ZO-1 drives TJ barrier formation (33).

**Figure 3:**
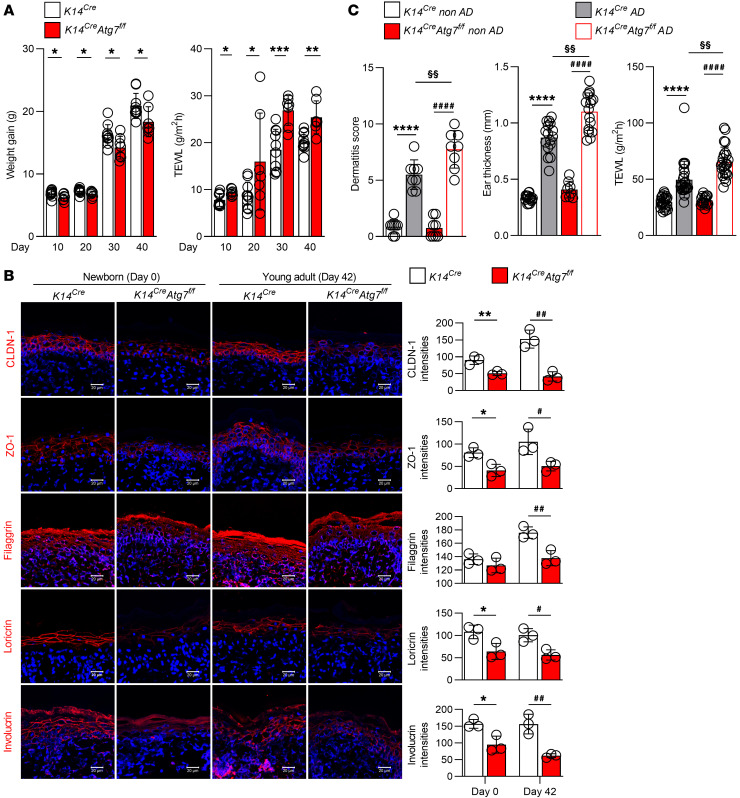
Keratinocyte-specific deficiency of autophagy exacerbates AD. (**A**) Body weight (left) and TEWL (right) of *K14^Cre^* mice and *K14^Cre^ Atg7^fl/fl^* mice from day 10 to day 40. (**B**) Representative immunofluorescence images (left) and quantification of the indicated proteins (right) from newborn mice and young adult mice at day 42; *n* = 3 per group. Scale bars: 20 μm. (**C**) Evaluation of dermatitis score, ear thickness, and TEWL in mouse ears and backs on day 19. *P < 0.05, **P < 0.01, ***P < 0.001, ****P < 0.0001, ^#^*P* < 0.05, ^##^*P* < 0.01, ^####^*P* < 0.0001, §§ *P* < 0.01. Statistical significance was determined by 2-tailed Student’s *t* test. All of the data are representative of 3 independent experiments.

To identify the role of autophagy in the regulation of the skin barrier in AD, we crossed *Atg7*-floxed mice (referred to as *Atg7^fl/fl^* mice hereafter) (34) with *K14-Cre* transgenic mice to generate mice with selective ablation of Atg7 in keratinocytes (referred to as *K14^Cre^ Atg7^fl/fl^* mice hereafter). We first confirmed the keratinocyte-specific deletion of autophagy in *K14^Cre^ Atg7^fl/fl^* mice, as evidenced by the absence of Atg7 (Supplemental Figure 3E) and LC3 (Supplemental Figure 3F, left) and the accumulation of p62 (Supplemental Figure 3F, right). Interestingly, from day 10 to day 40 postnatally, the weight gain of *K14^Cre^ Atg7^fl/fl^* mice was significantly less than that of *K14^Cre^* mice, and transepidermal water loss (TEWL) was higher in *K14^Cre^* *Atg7^fl/fl^* mice (Figure 3A). Furthermore, skin sections at both newborn age (day 0) and young adult age (day 42) showed downregulation of claudin-1, ZO-1, filaggrin, loricrin, and involucrin in *K14^Cre^* *Atg7^fl/fl^* mice (Figure 3B). Moreover, we established a DNCB-induced AD-like murine model using both *K14^Cre^* mice and *K14^Cre^* *Atg7^fl/fl^* mice and observed that *K14^Cre^* *Atg7^fl/fl^* AD mice displayed more severe inflammatory symptoms in the skin lesions than *K14^Cre^* AD mice (Supplemental Figure 3G). This was further confirmed by the finding that *K14^Cre^* *Atg7^fl/fl^* AD mice showed increased dermatitis scores, ear thickness, and TEWL compared with *K14^Cre^* AD mice (Figure 3C), suggesting that autophagy deficiency in keratinocytes may favor an AD-like phenotype.

### hBD-3–induced autophagy improves TJ barrier function in keratinocytes through aryl hydrocarbon signaling.

**Figure 4:**
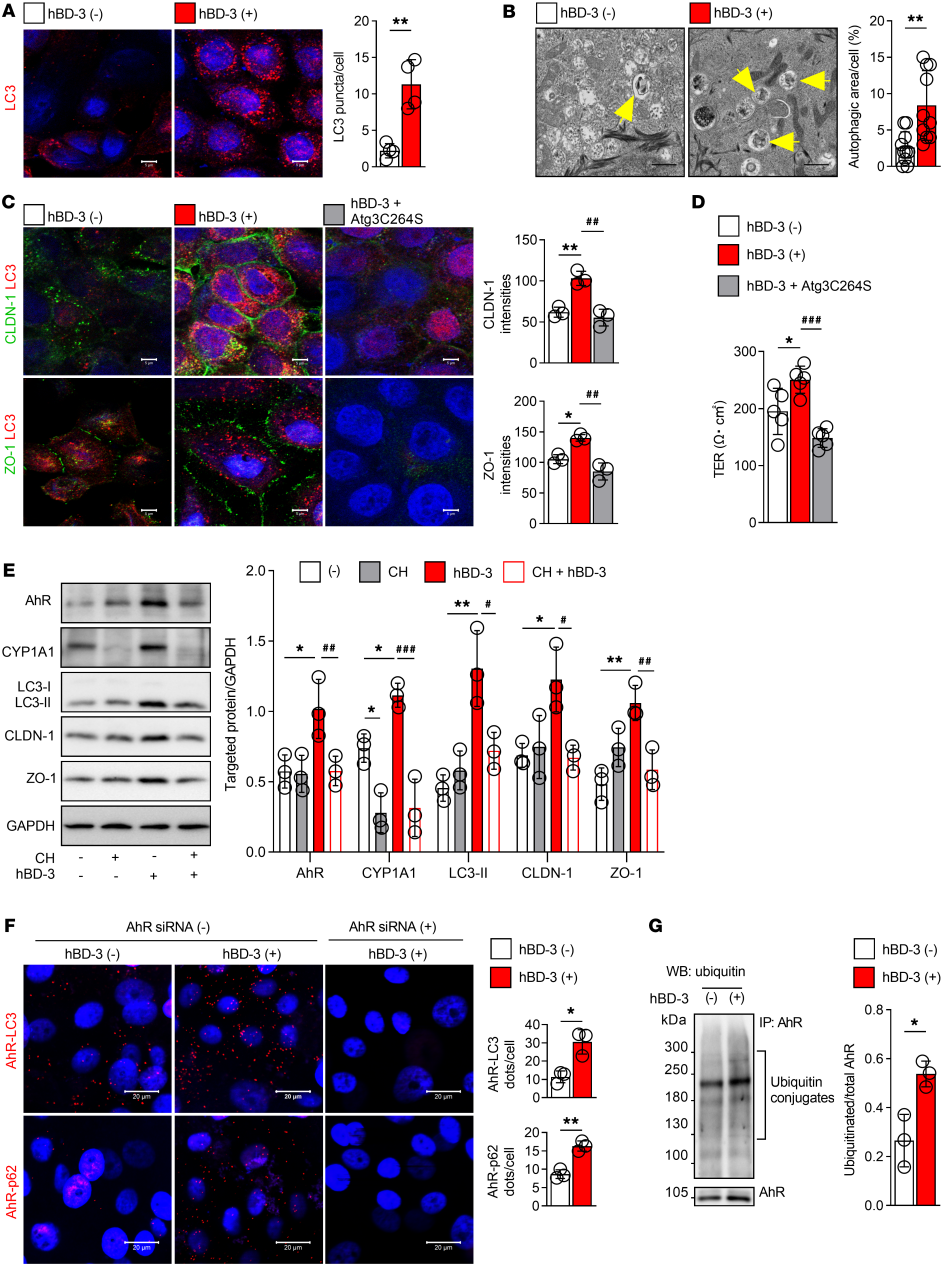
hBD-3–induced autophagy improves TJ barrier function in keratinocytes through AhR signaling. (**A** and **B**) Keratinocytes were treated with 10 μg/mL hBD-3 or vehicle control (–) for 9 hours. (**A**) Representative immunofluorescence images (left) and quantification of LC3 puncta (right); *n* = 4 per group. Scale bars: 5 μm. (**B**) Representative electron microscopic images (left) and quantification of autophagic areas (right); *n* = 10 per group. Scale bars: 10 μm. The yellow arrowheads indicate autophagic vacuoles. (**C**) Keratinocytes were transfected with adenoviruses carrying Atg3 or mutant Atg3C264S for 48 hours and then treated with 10 μg/mL hBD-3 for 9 hours. Representative immunofluorescence images (left) and quantification of claudin-1 and ZO-1 (right); *n* = 3 per group. Scale bars: 5 μm. (**D**) Keratinocyte layers grown on Transwell inserts were transfected with adenoviruses carrying Atg3 or mutant Atg3C264S for 48 hours and then treated with 10 μg/mL hBD-3 for 48 hours, and transepithelial electrical resistance (TER) was assessed by CellZscope. (**E**) Cells were pretreated with CH-223191 (CH) for 2 hours and then treated with 10 μg/mL hBD-3 for 9 hours. Representative immunoblots of the indicated proteins are shown. Quantification of the band intensities is shown in the right panel; *n* = 3 per group. (**F** and **G**) Keratinocytes were treated with 10 μg/mL hBD-3 or 0.01% acetic acid as a vehicle control for 9 hours. (**F**) Representative proximity ligation assay images (left) and quantification (right) of the AhR-LC3 and AhR-p62 interactions in keratinocytes with or without AhR siRNA transfection; *n* = 3 per group. Scale bars: 20 μm. (**G**) Representative Western blot images (left) and quantification of the band intensities (right) of AhR ubiquitination; *n* = 3 per group. Mean ± SD. *P < 0.05, **P < 0.01, ^#^*P* < 0.05, ^##^*P* < 0.01, ^###^*P* < 0.01. Statistical significance was determined by 2-tailed Student’s *t* test (**A**, **B**, **F**, and **G**) and 1-way ANOVA with Tukey’s multiple-comparison test (**C**–**E**). All of the data are representative of 3 independent experiments.

hBD-3 has been reported to improve TJ barrier function in human keratinocytes (35). Here, we asked whether hBD-3 may regulate the TJ barrier through the activation of keratinocyte autophagy. Among the 4 types of hBDs that are expressed in human skin (hBD-1 to hBD-4) (22), we observed that only hBD-3 significantly increased the amount of LC3-II, while hBD-1 and hBD-4 had no effect, and hBD-2 decreased the levels of LC3-II (Supplemental Figure 4A). We used E&P to inhibit autophagosome-lysosome fusion and observed that hBD-3 further increased the LC3-II levels in both the presence and absence of E&P, indicating that hBD-3 promoted active autophagic flux in keratinocytes (Supplemental Figure 4B). We also observed increases in the number of LC3-positive puncta in hBD-3–stimulated keratinocytes (Figure 4A), and electron microscope ultrastructural analysis showed that the number of autophagic vacuoles per cell was significantly higher in hBD-3–stimulated keratinocytes than in nonstimulated control cells (Figure 4B). These results suggest that hBD-3 increased the number of autophagosomes in keratinocytes.

We next sought to determine whether hBD-3–induced autophagy played a role in the regulation of TJ proteins in keratinocytes. hBD-3 increased the intercellular distribution of both claudin-1 and ZO-1, and treatment of keratinocytes with autophagy inhibitors, such as E&P, CQ, bafilomycin A1, and wortmannin, noticeably reduced hBD-3–induced claudin-1 and ZO-1 accumulation (Supplemental Figure 4C). Alternatively, hBD-3 failed to enhance the intercellular distribution of claudin-1 and ZO-1 in autophagy-deficient keratinocytes (Figure 4C), demonstrating that hBD-3–mediated regulation of epidermal TJs is associated with the activation of keratinocyte autophagy. This result was further confirmed by the finding that hBD-3–mediated improvement of transepithelial electrical resistance, a parameter used for the assessment of TJ barrier function, was abolished in Atg3C264S-transfected keratinocytes (Figure 4D) and reduced to baseline levels in the presence of autophagy inhibitors (Supplemental Figure 4D).

To identify the functional pathways involved in hBD-3–mediated keratinocyte autophagy activation, we first analyzed the differences in gene expression between autophagy-deficient keratinocytes and normal keratinocytes using DNA microarray assays and Ingenuity Pathway Analysis. While DNA microarrays detect mRNA transcripts (via the detection of cDNA), Ingenuity Pathway Analysis uses expression data to identify relevant functional pathways based on existing experimental databases. We found that aryl hydrocarbon (AhR) signaling, which is associated with the upregulation of skin barrier function (36), was the most affected pathway in autophagy-deficient keratinocytes (Supplemental Figure 5A), prompting us to ask whether AhR signaling may play a role in hBD-3–induced activation of keratinocyte autophagy. To test this hypothesis, we treated keratinocytes with an AhR antagonist (CH-223191), which significantly decreased CYP1A1, a cytochrome P450 enzyme induced following AhR activation, thus confirming that the AhR pathway was inhibited. Likewise, CH-223191 decreased hBD-3–induced expression of AhR, CYP1A1, LC3-II, claudin-1, and ZO-1, suggesting that hBD-3 induced autophagy and TJ proteins through the AhR pathway (Figure 4E).

mTOR is a key canonical regulator of autophagy in mammalian cells. Following nutrient limitation, mTORC1 signaling is turned off, leading to inhibition of cell growth and the activation of autophagy (7, 8). Although hBD-3 inhibited the phosphorylation of mTOR and its substrate S6K, pretreatment of keratinocytes with an AhR antagonist further enhanced the hBD-3–mediated inhibitory effect (Supplemental Figure 5B), suggesting that hBD-3–induced AhR-related autophagy was mTOR independent. In addition, the mitogen-activated protein kinase (MAPK) pathway reportedly acts as a bridge linking autophagy and inflammation (37, 38). Investigation of the relationship between MAPK and AhR signaling revealed that hBD-3 increased the phosphorylation of extracellular signal–regulated kinase, c-Jun N-terminal kinase, and p38; interestingly, however, only p38 phosphorylation was suppressed in the presence of the AhR antagonist (Supplemental Figure 5C). To further confirm the involvement of the p38 pathway in hBD-3–induced autophagy, cells were pretreated with a p38 inhibitor (SB203580) before stimulation with hBD-3. Surprisingly, the p38 inhibitor did not affect hBD-3–mediated autophagy (Supplemental Figure 5D), indicating that hBD-3–induced AhR-related autophagy is MAPK independent.

To further understand the association between AhR and hBD-3–mediated autophagy, the structure of the AhR protein was analyzed. Interestingly, we observed an LC3-interacting region–like (LIR-like) motif in the AhR protein sequence, as previously reported (39, 40) (Supplemental Table 4). This LIR motif allows autophagy receptors and adaptors to interact with LC3, followed by autophagosome formation and protein degradation in autolysosomes (41). Therefore, we hypothesized that following hBD-3 stimulation, AhR may interact with LC3 to promote the autophagy process. To verify this hypothesis, we used the proximity ligation assay, which permits the detection of protein-protein interactions in situ at the endogenous protein level. With this method, hBD-3 was found to increase AhR-LC3 ligation and AhR-p62 ligation in keratinocytes transfected with control siRNA but not in keratinocytes transfected with AhR siRNA (Figure 4F). Since p62 is a well-known ubiquitin-binding cargo receptor (42), we next examined the ubiquitination of AhR in hBD-3–treated keratinocytes and observed that AhR was ubiquitinated in hBD-3–stimulated keratinocytes, thus indicating that AhR was subsequently degraded by the ubiquitin-proteasome system, which may promote crosstalk between hBD-3 and autophagy (Figure 4G). Taken together, these observations imply that hBD-3–mediated autophagy is associated with AhR ubiquitination in keratinocytes.

### Mouse β-defensin-14 improves AD symptoms in mice.

To test whether hBD-3–induced autophagy activation might improve TJ barrier function in AD skin, we established an in vitro AD-like keratinocyte model by treating keratinocytes with IL-4 and IL-13. The LC3-II amounts (Supplemental Figure 6A) and intercellular distribution of claudin-1 and ZO-1 (Supplemental Figure 6B) were reduced in keratinocytes treated with a combination of IL-4 and IL-13, while the addition of hBD-3 reversed this effect. Interestingly, the expression of both claudin-1 and TJP1 (ZO-1) was abolished in autophagy-deficient keratinocytes (Supplemental Figure 6C), once again implying that hBD-3–induced autophagy is associated with the TJ barrier.

**Figure 5:**
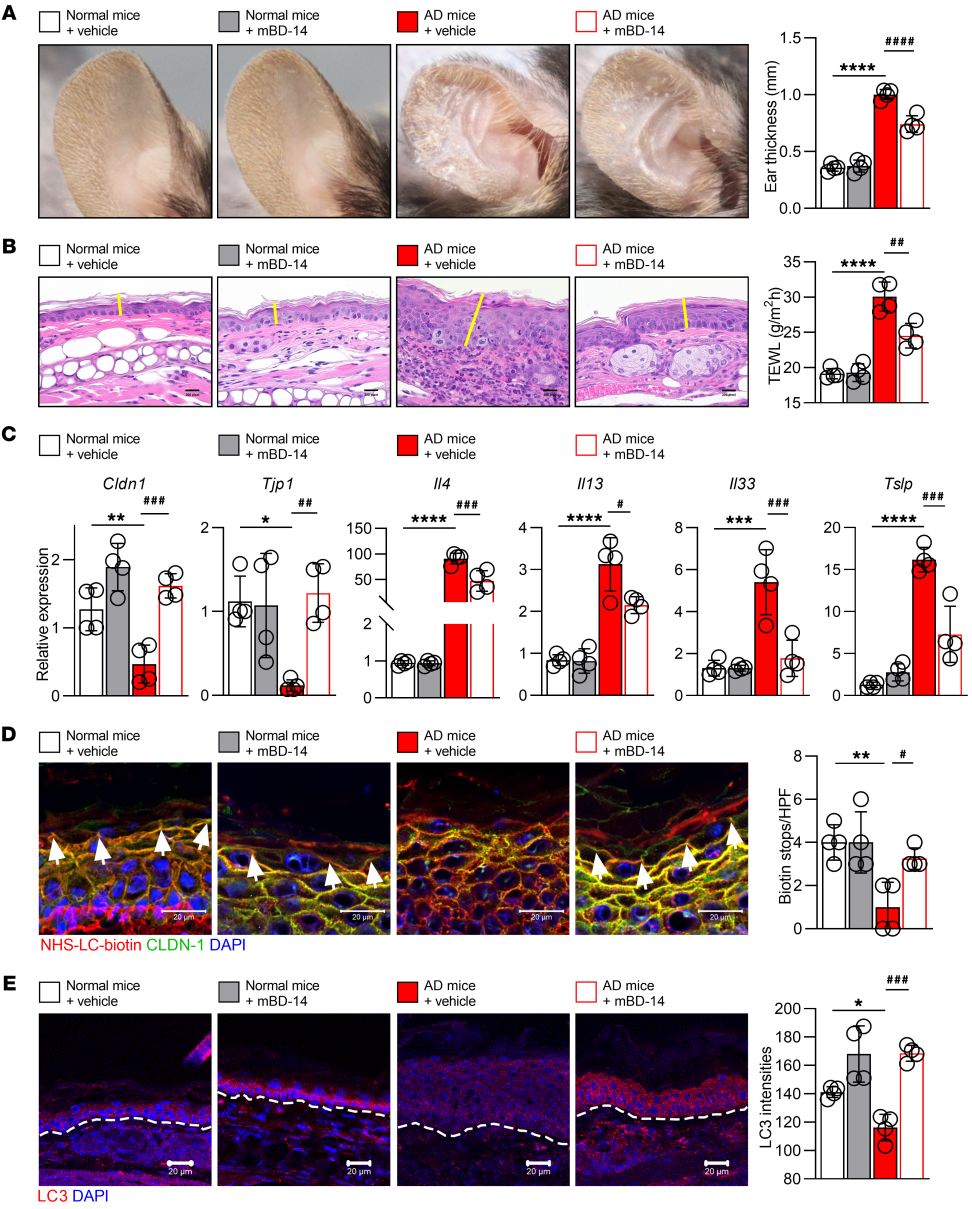
mBD-14 improves the symptoms of AD mice. (**A**) Representative images of ears from mice (left) and quantification of ear thickness (right); *n* = 4 per group. (**B**) Representative histological sections of mouse ears stained with H&E (left) and the TEWL of the mouse ears on day 19 (right). The yellow lines indicate the epidermis. Scale bars: 20 μm. (**C**) Real-time PCR analysis of the indicated genes in mouse ear samples. (**D**) Representative immunofluorescence images (left) and quantification of biotin tracer stops indicated by white arrowheads in the mouse skin (right); *n* = 4 per group. Scale bars: 20 μm. (**E**) Representative immunofluorescence images (left) and quantification of LC3 intensities in the mouse epidermis (right). The white dashed line indicates the basement membrane between the epidermis and dermis; *n* = 4 per group. Scale bars: 20 μm. Mean ± SD. *P < 0.05, **P < 0.01, ***P < 0.001, ****P < 0.0001, ^#^*P* < 0.05, ^##^*P* < 0.01, ^###^*P* < 0.001, ^####^*P* < 0.0001. Statistical significance was determined by 1-way ANOVA with Tukey’s multiple-comparison test. All of the data are representative of 3 independent experiments.

To verify whether hBD-3 improves the TJ barrier in AD through autophagy activation, mouse β-defensin-14 (mBD-14), a mouse ortholog of hBD-3, was subcutaneously injected into the lesions of AD mice (Supplemental Figure 7A). AD mice displayed significantly increased dermatitis scores, ear thickness, scratching behavior, TEWL, and total IgE, and importantly, these AD characteristics were noticeably reduced following mBD-14 treatment (Figure 5, A and B, and Supplemental Figure 7B). Moreover, the expression of *Cldn1* and *Tjp1* was significantly decreased, while that of the Th2 cytokines *Il4*, *Il13*, *Il33*, and *Tslp* and itch-related genes such as *Il31* and nerve growth factor was increased, in AD skin lesions. Strikingly, the administration of mBD-14 to AD mice recovered the expression of *Cldn1* and *Tjp1* and lowered the expression of Th2 cytokines (Figure 5C) and itch-related genes (Supplemental Figure 7C). Administration of mBD-14 to the skin lesions of AD mice also markedly decreased the number of CD4^+^ T cells (Supplemental Figure 7D) and tended to reduce the mast cell number, although this effect was not significant (Supplemental Figure 7E).

To study the role of mBD-14 in the regulation of TJ barrier function in AD mice, an NHS-LC-biotin tracer was injected into the lesional skin as well as normal skin as described in previous studies (32, 43). In normal mice with intact skin barrier function, the tracer does not penetrate up to the outermost layer of the epidermis, whereas in impaired TJ barriers, the tracer penetrates easily. As shown in Figure 5D, we observed that in the normal mice, the penetration of the tracer (red) into the epidermis was stopped (arrowheads) by the intact TJ barrier (represented by colocalization of claudin-1 and biotin tracer), whereas in AD mice, the tracer penetrated and passed through the epidermis, as shown by the decreased number of biotin stops compared with those in normal mice. Importantly, following mBD-14 administration, tracer penetration was stopped by the TJ barrier again, indicating that mBD-14 administration restored TJ barrier function. Interestingly, mBD-14 also recovered the expression of LC3 in the lesional skin of AD mice (Figure 5E), demonstrating that mBD-14–mediated improvements in AD mice may be associated with autophagy regulation.

### Autophagy is required for mBD-14–mediated improvements in AD mice.

**Figure 6:**
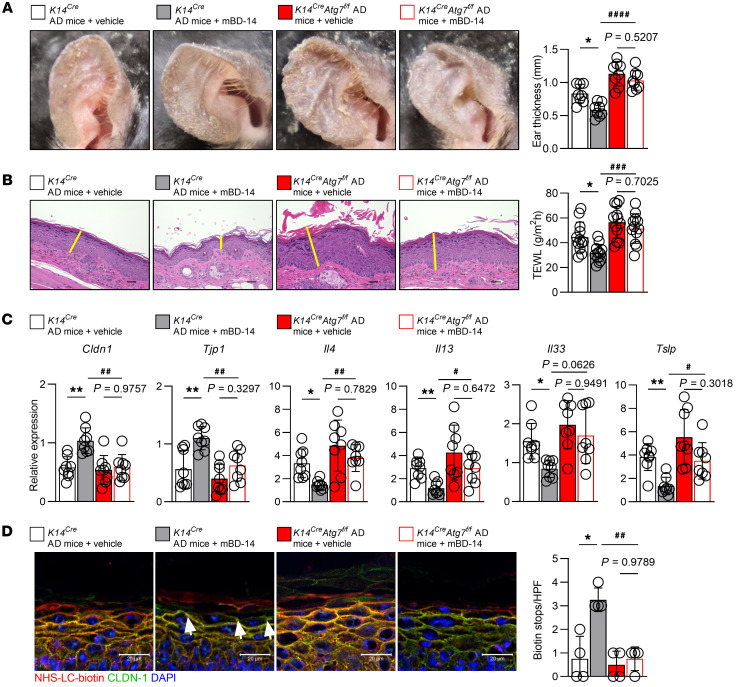
Autophagy is required for mBD-14–mediated improvement in AD mice. (**A**) Representative images of mouse ears (left) and quantification of ear thickness (right). (**B**) Representative histological sections of mouse ears stained with H&E (left) and TEWL of mouse ears on day 19 (right). The yellow lines indicate the epidermis. Scale bars: 20 μm. (**C**) Real-time PCR analysis of the indicated genes in mouse ear samples. (**D**) Representative immunofluorescence images (left) and quantification of biotin tracer stops indicated by white arrowheads in the mouse skin (right); *n* = 4 per group. Scale bars: 20 μm. Mean ± SD. *P < 0.05, **P < 0.01, ^#^*P* < 0.05, ^##^*P* < 0.01, ^###^*P* < 0.001, ^####^*P* < 0.0001. Statistical significance was determined by 1-way ANOVA with Tukey’s multiple-comparison test. All of the data are representative of 3 independent experiments.

To further elucidate the therapeutic role of hBD-3–mediated autophagy in AD skin, *K14^Cre^* AD mice and *K14^Cre^ Atg7^fl/fl^* AD mice were treated with mBD-14. While mBD-14 significantly decreased the ear thickness, TEWL, dermatitis scores, scratching behavior, and total IgE of *K14^Cre^* AD mice, this peptide failed to improve these AD characteristics in autophagy-deficient *K14^Cre^ Atg7^fl/fl^* AD mice (Figure 6, A and B, and Supplemental Figure 8A). Likewise, mBD-14 significantly increased the expression of *Cldn1* and *Tjp1* (*ZO-1*) and decreased the expression of *Il4*, *Il13*, *Il33*, and *Tslp* in *K14^Cre^* AD mice; however, these therapeutic effects were not observed in *K14^Cre^ Atg7^fl/fl^* AD mice (Figure 6C). More importantly, TJ barrier function was improved by mBD-14 in *K14^Cre^* AD mice but not *K14^Cre^ Atg7^fl/fl^* AD mice (Figure 6D). Taken together, these observations indicate that active autophagy is required for the mBD-14–mediated improvement of inflammatory responses and barrier function in AD mice.

### AhR signaling is required for mBD-14–mediated improvements in AD mice.

**Figure 7:**
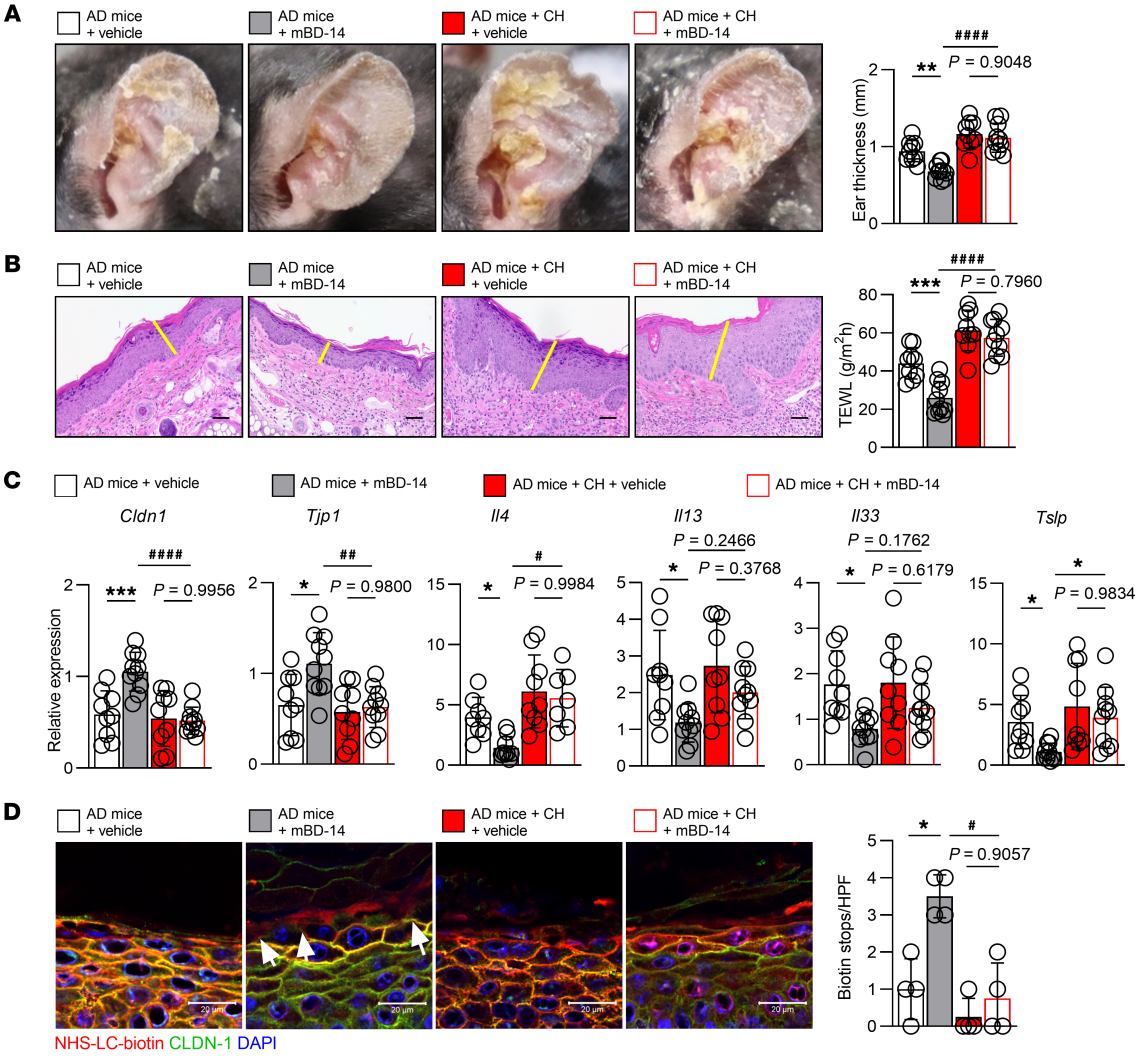
AhR signaling is required for mBD-14–mediated improvement in AD mice. (**A**) Representative images of mouse ears (left) and quantification of ear thickness (right). (**B**) Representative histological sections of mouse ears stained with H&E (left) and TEWL of mouse ears on day 19 (right). The yellow lines indicate the epidermis; *n* = 8 per group. Scale bars: 20 μm. (**C**) Real-time PCR analysis of the indicated genes in mouse ear samples; *n* = 8 per group. (**D**) Representative immunofluorescence images (left) and quantification of biotin tracer stops indicated by white arrowheads in the mouse skin (right); *n* = 4 per group. Scale bars: 20 μm. Mean ± SD. *P < 0.05, **P < 0.01, ***P < 0.001, ^#^*P* < 0.05, ^##^*P* < 0.01, ^####^*P* < 0.0001. Statistical significance was determined by 2-way ANOVA with Tukey’s multiple-comparison test. All of the data are representative of 3 independent experiments.

To further understand the role of AhR signaling in the mBD-14–mediated therapeutic effects in AD mice, the AhR antagonist CH-223191 was orally administered to mice to inhibit the effect of AhR (Supplemental Figure 8B). A modification of this method has been used to inhibit the effect of AhR in the liver (44). In our model, we confirmed that the expression of AhR in the epidermis was markedly decreased in the presence of CH-223191 (Supplemental Figure 8C); simultaneously, the expression of LC3 was decreased and that of p62 was increased in the epidermis by CH-223191 (Supplemental Figure 8D). We then established an AD murine model using AhR-inhibited mice; we observed that AD mice given CH-223191 showed no recovery of ear thickness, TEWL, and dermatitis scores, scratching behavior, or total IgE levels after treatment with mBD-14 (AD mice + CH + mBD-14), although these parameters were recovered in mBD-14–treated AD mice in the absence of CH-223191 (AD mice + mBD-14; Figure 7, A and B, and Supplemental Figure 8E). Similarly, as shown in Figure 7C, in the presence of CH-223191, mBD-14 did not increase *Cldn1* and *Tjp1* (*ZO-1*) expression. In addition, this peptide failed to decrease the expression of *Il4*, *Il13*, *Il33*, and *Tslp* in AhR-inhibited AD mice. Moreover, the administration of CH-223191 led to the failure of mBD-14 to improve TJ barrier function in AD mice, thus indicating that AhR is required for the mBD-14–mediated improvements in AD mice (Figure 7D).

## Discussion

Studies on the immunopathogenesis of AD have outlined the indispensable role of a dysfunctional epidermal barrier in the induction and substantial amplification of AD inflammation (2). Although autophagy contributes to the maintenance of the intestinal TJ barrier (24) and the regulation of epidermal differentiation (18, 45), the precise nature of the autophagy effect on the AD skin barrier remains poorly understood. Recently, it was reported that autophagy activation induced by the antimicrobial peptide LL-37 promoted intracellular killing of *Mycobacterium tuberculosis* in macrophages (46) and contributed to the elimination of *Porphyromonas gingivalis* internalized in keratinocytes (47), while S100A7-mediated downregulation of autophagy was associated with lipopolysaccharide-induced mitochondrial dysfunction in the keratinocyte cell line HaCaT (48). Moreover, the accumulation of hBD-3 is accompanied by LC3 in muscle fibers (49). Taken together, these findings prompted us to investigate the relationship between hBD-3 and autophagy and the effect of autophagy on the TJ barrier in keratinocytes.

Autophagy plays a substantial role in the homeostasis of skin development. Loss or impairment of autophagy has been associated with a number of skin conditions (50). For instance, the downregulation of LC3 (51) and the upregulation of p62 (52), which reflect a blockage of autophagy, are involved in inflammation in psoriasis; likewise, the autophagy activator rapamycin alleviates imiquimod-induced psoriasis-like dermatitis (51). However, conversely, one study showed that autophagy-related proteins are functionally active in the psoriatic epidermis (38). The multifactorial nature of psoriasis may be one of the reasons for these contradictory observations. In addition to psoriasis, the suppression of autophagy in sebaceous glands has been associated with the induction of acne inflammatory responses (53). This finding is in accordance with the fact that pharmacological inhibition of autophagy leads to the accumulation of sebaceous lipids (53).

Here, our study showed that LC3 levels and autophagic vesicles, the “gold standard” autophagy markers, were decreased in the epidermis of patients with AD as well as in different types of AD murine models compared with normal healthy participants or normal mice. Moreover, p62, another frequently used autophagy-related marker, was increased in the AD epidermis, consistent with previous studies (6, 14). The treatment of human keratinocytes with IL-4 and IL-13 has been used to mimic the features of AD in vitro (29). We observed that a combination of IL-4 and IL-13 noticeably inhibited autophagic flux, indicating that autophagy might be inactivated in keratinocytes of AD skin lesions. A previous study also reported that both IL-4 and IL-13 suppress autophagic flux in macrophages (54).

Recently, a few studies reported a crucial role of autophagy in epidermal differentiation (16–18, 45); however, it is still unknown whether autophagy contributes to the regulation of the epidermal barrier, an essential component in AD prevention. Epidermal barrier function largely relies on the SC barrier; perturbation of the SC barrier by tape stripping or acetone treatment has been shown to trigger the release of Th2 cytokines such as TSLP and IL-33, leading to subsequent skin inflammation (55–57). Filaggrin, an intracellular component of the SC, is assumed to be critical for normal cornification (2), as filaggrin-deficient mice exhibit reduced SC barrier function and spontaneous AD-like dermatitis (58, 59).

In addition to the SC, TJs are indispensable for the integrity of the skin barrier. TJs comprise transmembrane proteins, including claudin-1, and cytosolic scaffold proteins, including ZO-1. In claudin-1–knockout mice, epidermal TJs lose their tightness, and although these mice do not show any abnormalities in SC components, their SC displays compact hyperkeratosis and an increased TEWL, leading to their death at 24 hours after birth (32). Furthermore, the phase separation of ZO-1 drives the formation of epidermal TJs (33); epidermal TJ formation is delayed in ZO-1–deficient mice (60), implying the indispensable role of claudin-1 and ZO-1 in the formation of functional epidermal TJs. More importantly, in a murine AD-like model, TJ proteins were suppressed in skin inflammation but not directly affected by filaggrin deficiency (61). Here, autophagy inhibition/deficiency caused the downregulation of SC barrier–related proteins, including filaggrin, loricrin, and involucrin, and TJ barrier–related proteins, such as claudin-1 and ZO-1, and increased the TJ permeability and TEWL levels. To the best of our knowledge, this is the first report to demonstrate that inactivation of keratinocyte autophagy leads to defects in the skin barrier.

Selective autophagy maintains baseline levels of AhR (62), which was originally recognized as a transcription factor activated in response to environmental toxicants such as dioxins and polycyclic aromatic hydrocarbons (63). Depending on the ligand type, AhR ligation induces not only oxidative stress but also antioxidative responses (64, 65), which participate in a series of skin physiopathological processes, such as the photoinduced skin response and inflammation (66, 67). In addition, AhR signaling is essential for the coordinated upregulation of epidermal differentiation markers such as loricrin and involucrin, which are inhibited by Th2 cytokines, particularly in AD (68). Moreover, skin inflammation can be inhibited by AhR agonists, including tapinarof, an AhR ligand with antioxidative activity (69). Recent clinical trials have shown that the application of tapinarof is efficacious for patients with AD (70, 71).

Our microarray analysis revealed that the AhR pathway was the most affected signaling pathway in autophagy-deficient keratinocytes, suggesting that inhibition of AhR signaling might be one of the causes of skin barrier disruption in autophagy-deficient keratinocytes. We demonstrated that AhR signaling involved both hBD-3–mediated keratinocyte autophagy and improvement of the TJ barrier. Although both mTOR and MAPK are important pathways involved in the regulation of autophagy (7, 8, 37, 38), hBD-3–mediated AhR-related keratinocyte autophagy was mTOR and MAPK independent; however, this autophagy might be correlated with the ubiquitin-proteasome system, which is in line with a previous study (62). Thus, we herein provide evidence that hBD-3–induced AhR-related autophagy enhances epidermal barrier function.

Antimicrobial peptides (also known as host defense peptides), such as hBD-3, cathelicidin LL-37, and S100A7/psoriasin, exhibit not only a wide range of antimicrobial activities but also immunomodulatory effects, including inducing cell proliferation and differentiation, regulating the production of cytokines/chemokines, and improving barrier function in epidermal keratinocytes (35, 72). Compared with that seen in psoriasis, the expression of LL-37 and hBD-3 is decreased in patients with AD (73, 74). Although *S*. *aureus*, which frequently colonizes AD skin, can upregulate the expression of antimicrobial peptides (75), Th2 cytokines that are predominantly produced in AD skin downregulate this expression (76), while neutralization of Th2 cytokines restores it (23). hBD-3 contributes to both innate immunity and adaptive immunity in skin. Although hBD-3 has been shown to chemoattract and activate immune cells such as dendritic cells, T cells, and mast cells that may exacerbate AD (76–79), this peptide plays a key protective role in AD by killing various pathogens that colonize AD skin, improves epidermal barrier function, and controls cutaneous innervation (22, 35).

In summary, we demonstrated that dysfunctional autophagy plays a crucial role in generating epidermal barrier defects that sustain chronic inflammation in AD. Moreover, we highlighted the role of hBD-3 as a potentially novel autophagy activator in an approach to the treatment of AD that functions via autophagy activation, and uncovered the importance of AhR in hBD-3–mediated autophagy. We propose hBD-3 as a therapeutic target for the treatment of cutaneous diseases such as AD that are characterized by dysfunctional autophagy and skin barriers.

## Methods

### Animals.

*Atg7*-floxed mice (B6.Cg-Atg7<tm1Tchi>; RBRC02759) on a C57BL/6 background were generated as previously reported (34). *K14-Cre* mice (Tg[Krt14-cre]1Amc/J) were purchased from The Jackson Laboratory. C57BL/6 mice (aged 5–6 weeks) were purchased from Japan SLC Inc. (Tokyo, Japan). The mice were housed under specific pathogen–free controlled conditions with a 12-hour light/12-hour dark cycle and a steady temperature of 24°C ± 1°C and had ad libitum access to water and food. Genotyping was performed on day 10 postnatally by PCR assay with genomic DNA extracted from tail biopsies. The genotyping primers are provided in Supplemental Table 1.

### Human participants.

All patients were diagnosed based on Hanifin and Rajka’s diagnostic criteria. Biopsies of lesional skin were collected from 5 patients with AD, and biopsies of healthy skin were obtained from 5 healthy donors. Fresh skin samples were snap-frozen in liquid nitrogen and stored at −80°C for immunofluorescence analysis. The information of the participants without systemic therapy administration, including investigational agents used for over 4 weeks before study entry, was confirmed. Participants with a history of other autoimmune diseases, immune deficiency diseases, or tumors were excluded from the study.

### Primary normal human epidermal keratinocytes.

Primary normal human epidermal keratinocytes (FC-0007, Kurabo Industries) isolated from neonatal foreskins were cultured in serum-free HuMedia-KG2 keratinocyte growth medium (KK-2150S, Kurabo Industries) containing human epidermal growth factor (0.1 ng/mL), insulin (10 μg/mL), hydrocortisone (0.5 μg/mL), gentamicin (50 μg/mL), amphotericin B (50 ng/mL), and bovine brain pituitary extract (0.4%, vol/vol) at 37°C in a humidified atmosphere of 95% air and 5% CO_2_, as previously described (80). The cells grown up to 80% confluence were continually cultured in medium containing high (1.8 mM) Ca^2+^ for 24 hours to mimic keratinocytes of the second layer of stratum granulosum, where TJs are found (81).

### Generation of the in vitro AD model.

Human keratinocytes were stimulated with 100 ng/mL recombinant IL-4 (AF-200-04, PeproTech) and IL-13 (CYT-446) alone or in combination in HuMedia-KG2 keratinocyte growth medium containing gentamicin (50 μg/mL) to mimic the features of AD pathology as reported previously (29).

### Generation of the in vivo AD model.

Mice were sensitized and repeatedly challenged at the same skin site with DNCB to induce AD-like skin lesions as previously described (26). Briefly, the day before the application (day –5), the backs of the mice were shaved. The ears and backs of the mice were then treated with 1% DNCB once (day –4). Four days later, 0.4% DNCB was applied at the same site 3 times per week for 3 weeks (days 1–19). A total dermatitis score (maximum score 12) indicating the clinical severity was defined as the sum of the individual scores graded as 0 (none), 1 (mild), 2 (moderate), and 3 (severe), which were given for each of 4 symptoms (erythema/hemorrhage, scaling/dryness, edema, and excoriation/erosion). To minimize the variation, the investigators were blinded to the treatment conditions of the different groups when scoring for symptoms. Ear thickness was assessed by a micrometer (Mitutoyo). TEWL was measured by a Tewameter TM Nano (Courage + Khazaka electronic GmbH).

Furthermore, calcipotriol (MC903), a vitamin D_3_ analog, was dissolved in ethanol and topically applied to the ears and shaved dorsal skin of the mice to induce dermatitis as described previously (27). In addition, AD-like dermatitis was induced in NC/Nga mice (Japan SLC Inc.) by topical application of 150 μL of 4% SDS and 100 mg of *Dermatophagoides farinae* extract ointment on the shaved dorsal skin of the mice twice a week for 3 weeks as described previously (28).

### Treatment of mice.

The dorsal skin lesions and ears of the AD mice were subcutaneously injected with 100 μL and 25 μL of 10 μg/mL recombinant mBD-14 (CYT-945, Prospec) on days 15, 17, and 18. On day 19, the serum and skin biopsies were harvested and analyzed as described below.

### Histological analysis.

Mouse dorsal skin and ear tissues were fixed in 20% formalin neutral buffer solution, embedded in paraffin, sectioned, and stained with H&E for histopathological examination. Images were captured using a Zeiss microscope or Zeiss Axiocam 208 color camera (Carl Zeiss) and were analyzed with ImageJ software (version 1.52a, NIH). Histopathological evaluation of all skin sections was performed in a blinded fashion.

### Immunostaining analysis.

Tissue biopsies were directly embedded in OCT compound, and frozen sections were fixed in preheated 4% paraformaldehyde in PBS for 10 minutes. Sections were permeabilized with 0.01% Triton X-100 in PBS for 10 minutes, blocked with ImmunoBlock (CTKN001, KAC Co.) for 30 minutes, and then incubated overnight at 4°C with the appropriate primary antibodies. After incubation with secondary antibodies, samples were mounted using antifade mountant with NucBlue stain (P36981, Invitrogen). Images were processed using the ZEN 2011 software of the Zeiss Laser Scanning Microscope 780 system (Carl Zeiss). Quantification of the fluorescence intensities of the images was performed with ImageJ software. The antibodies used in this study are listed in Supplemental Table 2.

### TJ permeability assay.

A TJ permeability assay using the surface biotinylation technique was performed as previously described (43). Briefly, EZ-link Sulfo-NHS-LC-Biotin (21338, Thermo Fisher Scientific) in PBS containing 1 M CaCl_2_ was injected dermally into the ears and backs of the mice. After a 30-minute incubation, the skin was removed and immediately embedded in OCT compound. Frozen sections (6 μm) were subsequently fixed in 4% paraformaldehyde in PBS for 10 minutes, permeabilized with 0.01% Triton X-100 in PBS for 10 minutes, blocked with ImmunoBlock for 30 minutes, and then incubated overnight at 4°C with anti–claudin-1 antibody. After 3 washes with blocking buffer, the sections were incubated with a mixture of Alexa Fluor 488–conjugated goat anti-mouse antibody and streptavidin Alexa Fluor 594–conjugated antibody for 1 hour. After mounting, the images were processed as described above using a Zeiss Laser Scanning Microscope 780 system.

### Immunocytochemistry analysis.

Keratinocytes were plated on 12-mm-diameter coverslips. After the indicated treatments, the coverslips were fixed in preheated 4% paraformaldehyde in PBS for 10 minutes, quenched with NH_4_Cl for 10 minutes, permeabilized with 0.01% Triton X-100 in PBS for 5 minutes, blocked with ImmunoBlock at 4°C for 30 minutes, and then incubated overnight at 4°C with primary antibodies against LC3, p62, claudin-1, and ZO-1. After incubation, the cells were stained with Alexa Fluor 594–conjugated goat anti-rabbit antibody and Alexa Fluor 594– or 488–conjugated goat anti-mouse antibodies as the secondary antibodies. Images were analyzed with a Zeiss Laser Scanning Microscope 780 system, and quantification of the fluorescence intensities of the images was performed with ImageJ software. All of the antibodies used in this study are described in Supplemental Table 2.

### Transmission electron microscopy.

Mouse skin tissues or human keratinocytes were fixed overnight at 4°C in 2.5% (vol/vol) glutaraldehyde in 0.1 M PBS (pH 7.4) and postfixed in 1% (wt/vol) OsO_4_ in 0.1 M PBS. After dehydration through a graded series of ethanol, the skin tissues and keratinocytes were embedded in Epon 812 (Oken-Shoji). Ultrathin sections were serially cut by an ultramicrotome (model UC6, Leica) and placed on copper grids, followed by analysis with an H-600IV transmission electron microscope (Hitachi). The samples were processed under a transmission electron microscope (model HT7700, Hitachi).

### Inhibition experiments.

Keratinocytes were incubated with IL-4, IL-13, or hBD-3 in the absence or presence of E64d (4321-v, Peptide Institute Inc.) in combination with pepstatin A (4397-v, Peptide Institute Inc.) (E&P), chloroquine (C6628, MilliporeSigma), bafilomycin A1 (11038, Cayman Chemical), wortmannin (10010591, Cayman Chemical), or CH-223191 (16154, Cayman Chemical) for 2 hours.

### Adenovirus-mediated silencing of gene expression.

An adenovirus expression vector kit (Takara Bio) was used to prepare Atg3C264S and Atg3 adenoviruses as previously reported (31). To express exogenous Atg3C264S and Atg3 proteins, keratinocytes were plated in 6-well dishes in 2 mL of growth medium containing human epidermal growth factor, insulin, hydrocortisone, bovine brain pituitary extract, and 1.8 mM Ca^2+^ for 24 hours before infection, and the medium was replaced with fresh medium containing Atg3C264S or Atg3 adenoviruses. After 48 hours, the cells were treated with hBD-3, followed by immunoblotting or immunocytochemistry analysis.

### Total RNA extraction and real-time quantitative PCR.

Total RNA was extracted from keratinocytes and skin tissues using the RNeasy Plus Micro kit (74034, Qiagen) and RNeasy Plus Universal Mini kit (73404, Qiagen), respectively. Reverse transcription of 1 μg of total RNA to first-strand cDNA was performed using the ReverTra Ace qPCR RT Master Mix (FSQ-201, Toyobo) or ReverTra Ace qPCR RT Master Mix with gDNA remover (FSQ-301, Toyobo) according to the manufacturer’s instructions. Real-time PCR was performed using the QuantiTect SYBR Green PCR Kit (204145, Qiagen). Amplification and detection of mRNA were performed using the StepOnePlus Real-Time PCR System (Life Technologies) following the manufacturer’s specifications. The sequence-specific primer sets used in this study are listed in Supplemental Table 3. All real-time PCRs were performed in triplicate, and the fold changes in gene expression are reported relative to the values in the untreated controls.

### Western blot analysis.

The samples derived from human keratinocytes and mouse skin tissues were lysed with RIPA lysis buffer (9806, Cell Signaling Technology). Protein concentrations were determined using Precision Red Advanced Protein Assay reagent (ADV02, Cytoskeleton), and equal amounts of total protein were subjected to electrophoresis with 8%–15% SDS-PAGE gels followed by transfer to PVDF membranes (IPVH00010, Merck Millipore, Burlington, Massachusetts, USA). The membranes were then blocked in ImmunoBlock buffer for 1 hour at room temperature followed by overnight incubation at 4°C with primary antibodies according to the manufacturer’s instructions. Labeling of the primary antibodies was detected using sheep anti-rabbit or sheep anti-mouse antibodies conjugated to horseradish peroxidase (NA934 V and NA931 V, respectively; Amersham Biosciences), developed with the Luminata Forte Western horseradish peroxidase substrate (WBLUF0100, Merck Millipore, Billerica, Massachusetts, USA), and then imaged using Fujifilm LAS-4000 Plus. ImageJ was used for quantification of the band intensity in the images.

### ELISA.

To detect the levels of total IgE in the mouse serum, the sera were collected, and total IgE levels were evaluated as follows. Ninety-six-well plates were coated with 2 μg/mL purified rat anti-mouse IgE (553413, BD Biosciences) overnight at 4°C and blocked with 20% ImmunoBlock at 37°C for 90 minutes. Samples and purified mouse IgE (554118, BD Biosciences) used as standards were added to the assay wells and incubated at 37°C for 80 minutes. After incubation with horseradish peroxidase–conjugated anti-mouse IgE (LO-ME-2-HRP-1, Dianova), TMB substrate reagent (555214, BD Biosciences) was added to the wells for 20 minutes, and an equal volume of stop solution containing 1 M sulfuric acid was applied, followed by reading of the optical density at 450 nm.

### Measurements of transepithelial electrical resistance.

Transepithelial electrical resistance (TER) measurements were performed as described previously (35). Briefly, keratinocytes grown on 0.6 cm^2^ Transwell filters were transferred into 1.8 mM Ca^2+^ medium, and hBD-3 was added to both the apical and basal compartments in the absence or presence of various inhibitors. The TER across the keratinocyte monolayers was measured at 48 hours post-stimulation using CellZscope (NanoAnalytics). All experiments included DMSO at a concentration less than 0.1% as a vehicle.

### Transfection of siRNA.

The siRNA duplex sense sequences used for AhR were 5′-GGCUCUUUCAAGAUAGUAAtt-3′ and 5′-GCAUGAUAGUUUUCCGGCUtt-3′. The cells were transfected with 30 pmol of 2 different siRNA duplexes targeting AhR or the scrambled control siRNA (4390843, Invitrogen) using Lipofectamine RNAiMAX (Invitrogen) according to the manufacturer’s specifications. After 24 hours of transfection, the cells were subsequently stimulated with hBD-3.

### Proximity ligation assay.

Cells were plated on 12-mm-diameter glass coverslips and grown to approximately 80% confluence. The coverslips were fixed in preheated 4% paraformaldehyde in PBS for 10 minutes, quenched with NH_4_Cl for 10 minutes, permeabilized with 0.01% Triton X-100 in PBS for 5 minutes, and then blocked with Duolink blocking solution at 37°C for 60 minutes. After incubation with mouse anti-AhR (A-3) antibody, rabbit anti-LC3 and rabbit anti-p62 antibodies in Duolink antibody diluent were used for the interaction study at 4°C overnight. Ligation and amplification of the Duolink probe were performed according to the manufacturer’s recommendations. Coverslips were mounted, samples were observed using a Keyence BZ-X700 fluorescence microscope (Keyence), and the results were analyzed by ImageJ software.

### Immunoprecipitation and coimmunoprecipitation.

Cells were lysed with RIPA lysis buffer containing 3 deubiquitylase inhibitors — 1,10-phenanthroline (P0221, Tokyo Chemistry Industry), *N*-ethylmaleimide (E0136, Tokyo Chemistry Industry), and PR-619 (HY-13814, MedChemExpress) — for the immunoprecipitation experiments and the analysis of ubiquitylated proteins by anti-multiubiquitin antibody. One to two milligrams of whole-cell lysates were used for immunoprecipitation and coimmunoprecipitation of AhR using the anti-AhR antibody. Briefly, the anti-AhR antibody in (NH_4_)_2_SO_4_ was coupled to the pre-equilibrated Protein G Dynabeads in NaH_2_PO_4_ at 37°C overnight, following the manufacturer’s instructions. The AhR-coupled beads were then added to each sample and incubated on an orbital shaker at 0.5 *g* overnight at 4°C. After washing 3 times for 5 minutes each with the assay buffer, the beads were eluted with electrophoresis sample buffer for SDS-PAGE, followed by Western blot analysis.

### Microarray gene expression analysis.

Total RNA isolation and the evaluation of the quality and integrity of RNA were performed according to the user guide of the GeneChip WT Plus Reagent Kit Manual Target Preparation for GeneChip Whole Transcript Expression Arrays kit (Applied Biosystems). Briefly, 3 biological repeats were hybridized to the Clariom S Human Array (902927, Applied Biosystems). Robust multichip analysis normalization and fold change calculation of microarray data were performed with GeneSpring 14.9 software (Agilent Technology) to assess gene regulation. Genes with an estimated percentage of false-positive predictions less than 0.01 and a fold change of at least 1.5 were considered differentially expressed. Gene annotations were obtained from the NetAffx database (82). The microarray data were subsequently analyzed to identify the affected signaling pathways using Ingenuity Pathway Analysis (Ingenuity Systems, IPA Winter 2020 series). The microarray data were deposited in the NCBI’s Gene Expression Omnibus database (http://www.ncbi.nlm.nih.gov/gds; GEO GSE183921).

### Statistics.

All statistical analyses were performed with GraphPad Prism 9 software (version 9.0.0). 2-tailed student’s *t* test was used to compare 2 groups, and 1-way ANOVA with Tukey’s multiple-comparison test was used for comparisons of multiple groups. *P* values of less than 0.05 were considered statistically significant.

### Study approval.

All animal care procedures and experiments were approved by the Institutional Animal Care and Use Committee of Juntendo University Graduate School of Medicine (approval no. 2021255). The experimental procedures were conducted in accordance with the *Guide for the Care and Use of Laboratory Animals*, 8th edition (National Academies Press, 2011). All animal studies were reported according to the ARRIVE (Animal Research: Reporting In Vivo Experiments) guidelines for reporting experiments involving animals (83).

The present study adhered to the tenets of the Helsinki Declaration and was approved by the Ethics Committee of the Juntendo University Urayasu Hospital (Chiba, Japan) (approval no. 1-076). Written informed consent was obtained from the donors prior to the study.

## Author contributions

GP and FN conceived the project and designed the experiments. GP, ST, RI, and HLTN performed the biological and animal experiments. GP, ST, and RI performed the microarray data analysis. GP and FN analyzed the additional data and wrote the manuscript. MK, SI, and FN conceived and directed the project. RK, M Tominaga, and KT collected human skin biopsies. GP, ST, RI, HLTN, YU, JVTP, HY, M Takahashi, TO, RK, M Tominaga, KT, JK, SK, MK, KO, HO, SI, and FN reviewed and edited the manuscript. All authors read and agreed to the published version of the manuscript.

## Supplementary Material

Supplemental data

## Version 1 07/14/2022

In-Press Preview

## Version 2 09/01/2022

Electronic publication
