# Supplementary material for: Human β-defensin-3 attenuates atopic dermatitis–like inflammation through autophagy activation and the aryl hydrocarbon receptor signaling pathway
Source: J Clin Invest. 2022 Sep 1;132(17):e156501. doi: 10.1172/JCI156501 (PMC9435650; doi:10.1172/JCI156501)
Supplement: Supplemental data [file jci-132-156501-s122.pdf]

Human-β-defensin-3 attenuates atopic dermatitis-like inflammation through autophagy activation and the aryl hydrocarbon receptor signaling pathway

Ge Peng[1, 2], Saya Tsukamoto[1, 2], Risa Ikutama[1, 2], Hai Le Thanh Nguyen[1, 2], Yoshie Umehara[1], Juan V. Trujillo-Paez[1], Hainan Yue[1, 2], Miho Takahashi[1, 2], Takasuke Ogawa[2], Ryoma Kishi[3, 4], Mitsutoshi Tominaga[3], Kenji Takamori[3, 4], Jiro Kitaura[1], Shun Kageyama[5], Masaaki Komatsu[5], Ko Okumura[1], Hideoki Ogawa[1], Shigaku Ikeda[1, 2], François Niyonsaba[1, 6]

[1] Atopy (Allergy) Research Center, Juntendo University Graduate School of Medicine, 2-1-1 Hongo, Bunkyo-ku, Tokyo 113-8421, Japan.

[2] Department of Dermatology and Allergology, Juntendo University Graduate School of Medicine, 2-1-1 Hongo, Bunkyo-ku, Tokyo 113-8421, Japan.

[3] Juntendo Itch Research Center (JIRC), Institute for Environmental and Gender-Specific Medicine, Juntendo University Graduate School of Medicine, 2-1-1 Tomioka, Urayasu, Chiba 279-0021, Japan.

[4] Department of Dermatology, Juntendo University Urayasu Hospital, 2-1-1 Tomioka, Urayasu, Chiba 279-0021, Japan.

[5] Department of Physiology, Juntendo University Graduate School of Medicine, 2-1-1 Hongo, Bunkyo-ku, Tokyo 113-8421, Japan.

[6] Faculty of International Liberal Arts, Juntendo University, 2-1-1 Hongo, Bunkyo-ku, Tokyo 113-8421, Japan.

Address of correspondence: François Niyonsaba, Atopy (Allergy) Research Center and Faculty of International Liberal Arts, Juntendo University, 2-1-1 Hongo, Bunkyo-ku, Tokyo 113-8421, Japan; francois@juntendo.ac.jp; Tel.: +81-3-5802-1591; Fax: +81-3-3813-5512

Supplemental material

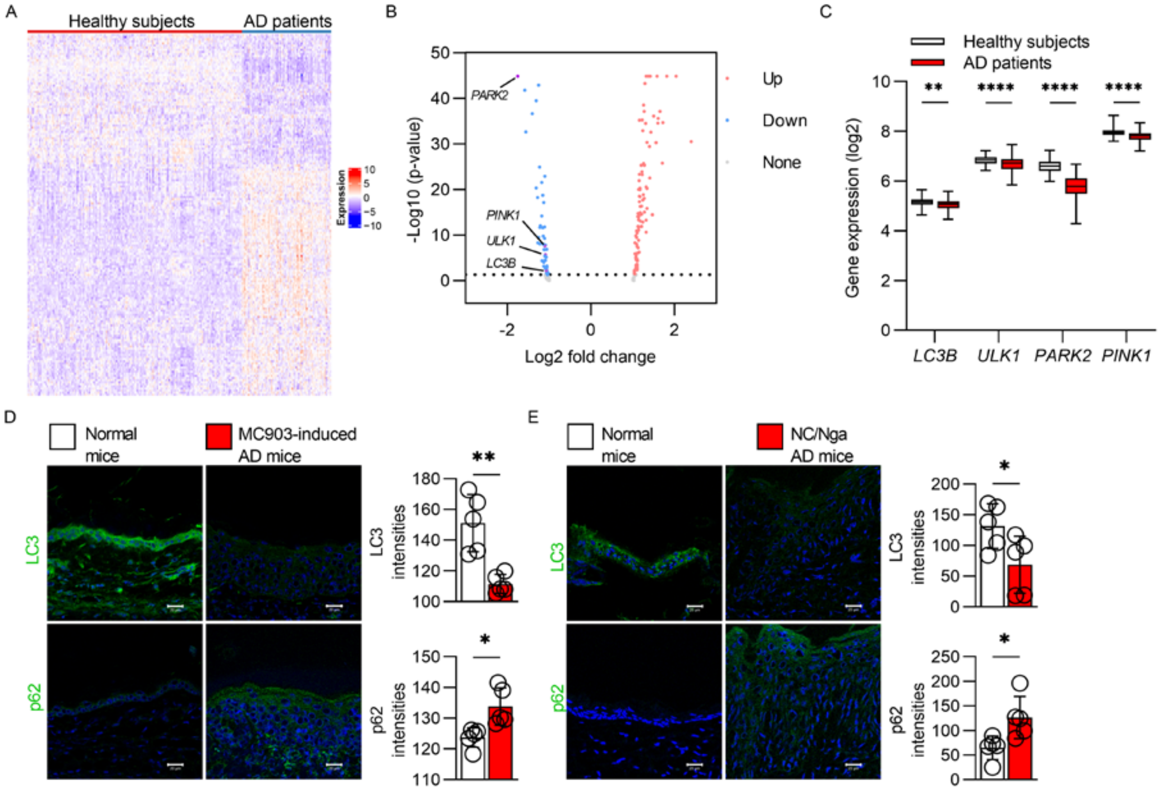

Figure S1. Autophagy-related proteins are dysregulated in AD skin lesions

(A) Heatmap of 202 autophagy-related genes differentially expressed between 84 patients with AD and 199 healthy volunteers. (B) Volcano plot of the differentially expressed autophagy-related genes. (C) Violin plot of the autophagy-related genes differentially expressed between 84 patients with AD and 199 healthy volunteers. (D) Immunofluorescence staining of LC3 and p62 in the epidermis of MC903-induced AD mice and normal mice. Representative immunofluorescence images of skin (left) and quantification of staining intensities in the epidermis (right). Scale bar: 5 μm, n = 5/group. (E) Immunofluorescence staining of LC3 and p62 in the epidermis of NC/Nga AD mice and normal mice. Representative immunofluorescence images of skin (left) and quantification of staining intensities in the epidermis (right). Scale bar: 5 μm, n = 5/group. *P <

0.05, **$P < 0.01$, ****$P < 0.0001$. Statistical significance was determined by two-tailed Student’s *t* test. All of the data are representative of three independent experiments.

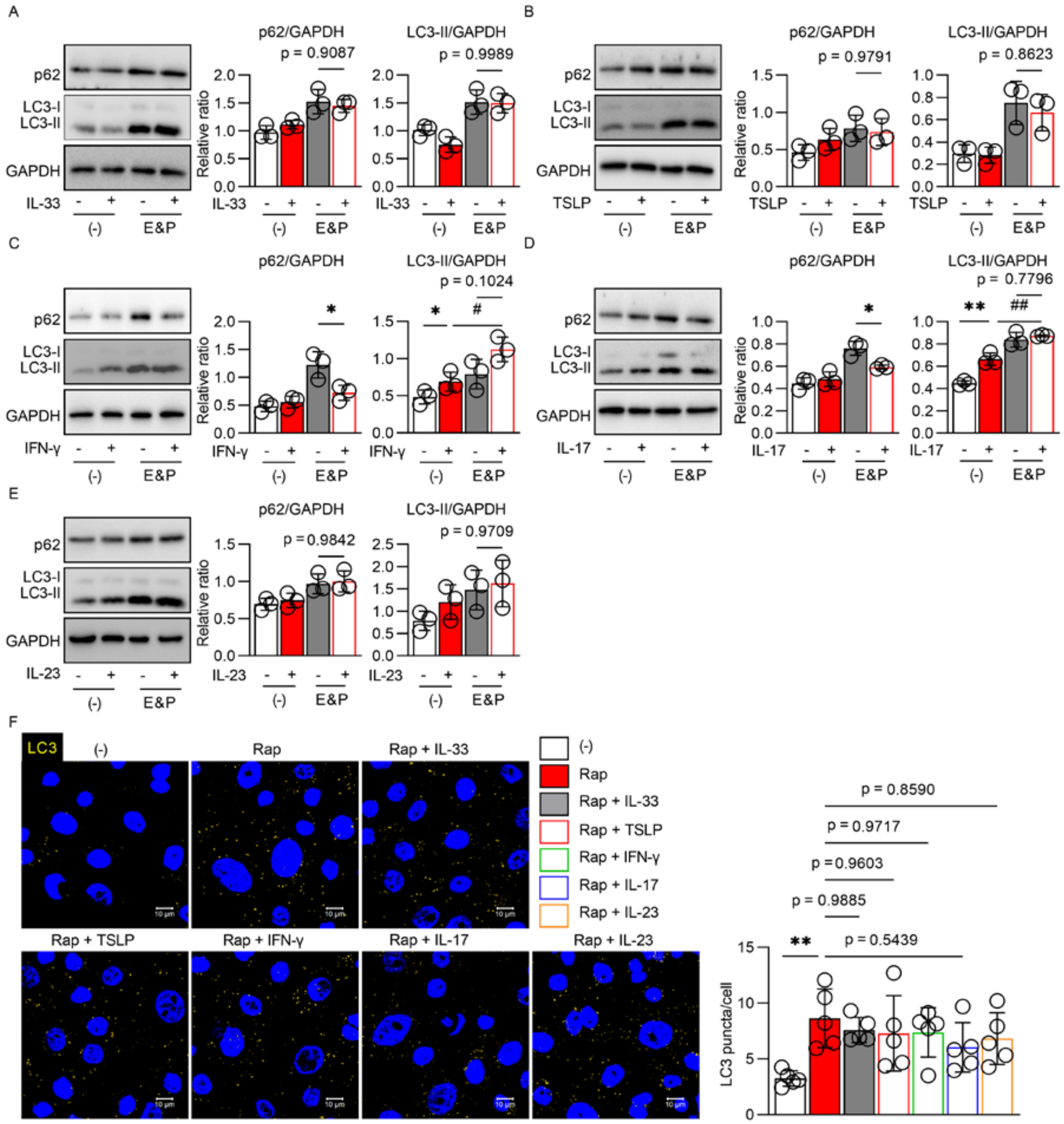

Figure S2. Effects of inflammatory cytokines on keratinocyte autophagy

(A-E) Keratinocytes were stimulated with (A) IL-33, (B) TSLP, (C) IFN-γ, (D) IL-17 or (E) IL-23 in the presence or absence of 10 μg/ml E&P for 12 hours. Representative LC3 and p62 immunoblots (left) and quantification of band intensities (right), n=3/group. (F) Keratinocytes were stimulated with or without IL-33, TSLP, IFN-γ, IL-17 or IL-23 in the presence of 10 μM rapamycin (Rap) for 12 hours, n = 3/group. Representative immunofluorescence images (right) and quantification of LC3 puncta (left). Scale bar: 10 μm. The data are presented as the mean ± SD. $^{*}P < 0.05$, $^{**}P < 0.01$, $^{\#}P < 0.05$, $^{\#\#}P < 0.01$. Statistical significance was determined by one-way ANOVA with Tukey's multiple comparisons test and two-tailed Student's *t* test. All of the data are representative of three independent experiments.

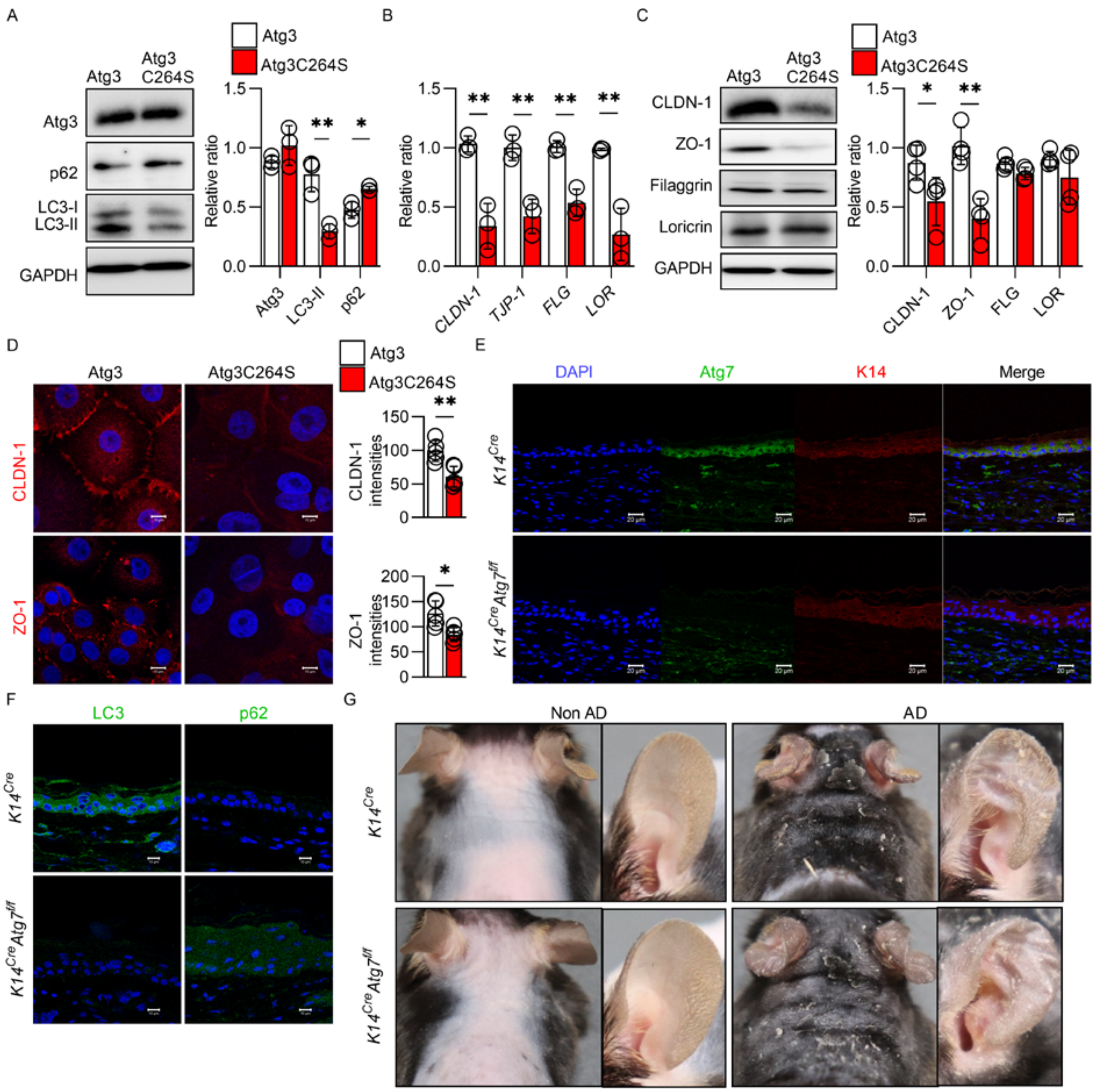

Figure S3. Keratinocyte-specific deficiency of autophagy exacerbates AD

(A-D) Keratinocytes were transfected with adenoviruses carrying mutant Atg3C264S or Atg3 for 48 hours. (A) Keratinocytes were transfected with adenoviruses carrying mutant Atg3C264S or Atg3 for 48 hours. Representative immunoblots (left) and quantification (right) of the indicated proteins are shown. GAPDH was used as a loading control, n= 3/group. (B) Real-time PCR analysis

of claudin-1 (*CLDN-1*), tight junction protein-1 (*TJP-1*), filaggrin (*FLG*) and loricrin (*LOR*) gene expression, n = 3/group. (C) Representative immunoblots of CLDN-1, *zonula occludens*-1 (ZO-1), filaggrin and loricrin. GAPDH was used as a loading control, n = 4/group. Quantification of band intensities is shown in right panels. (D) Representative immunofluorescence images (left) and quantification of CLDN-1 and ZO-1 (right), n = 5/group. Scale bar: 10 μm. (E) Immunofluorescence staining of Atg7 and K14 in the epidermis of $K14^{Cre}$ mice and $K14^{Cre}Atg7^{f/f}$ mice. (F) Immunofluorescence staining of LC3 and p62 in the epidermis of $K14^{Cre}$ mice and $K14^{Cre}Atg7^{f/f}$ mice. (G) Representative images of mouse ears and backs (right). $^{*}P < 0.05$, $^{**}P < 0.01$. Statistical significance was determined by two-tailed Student's *t* test. All of the data are representative of three independent experiments.

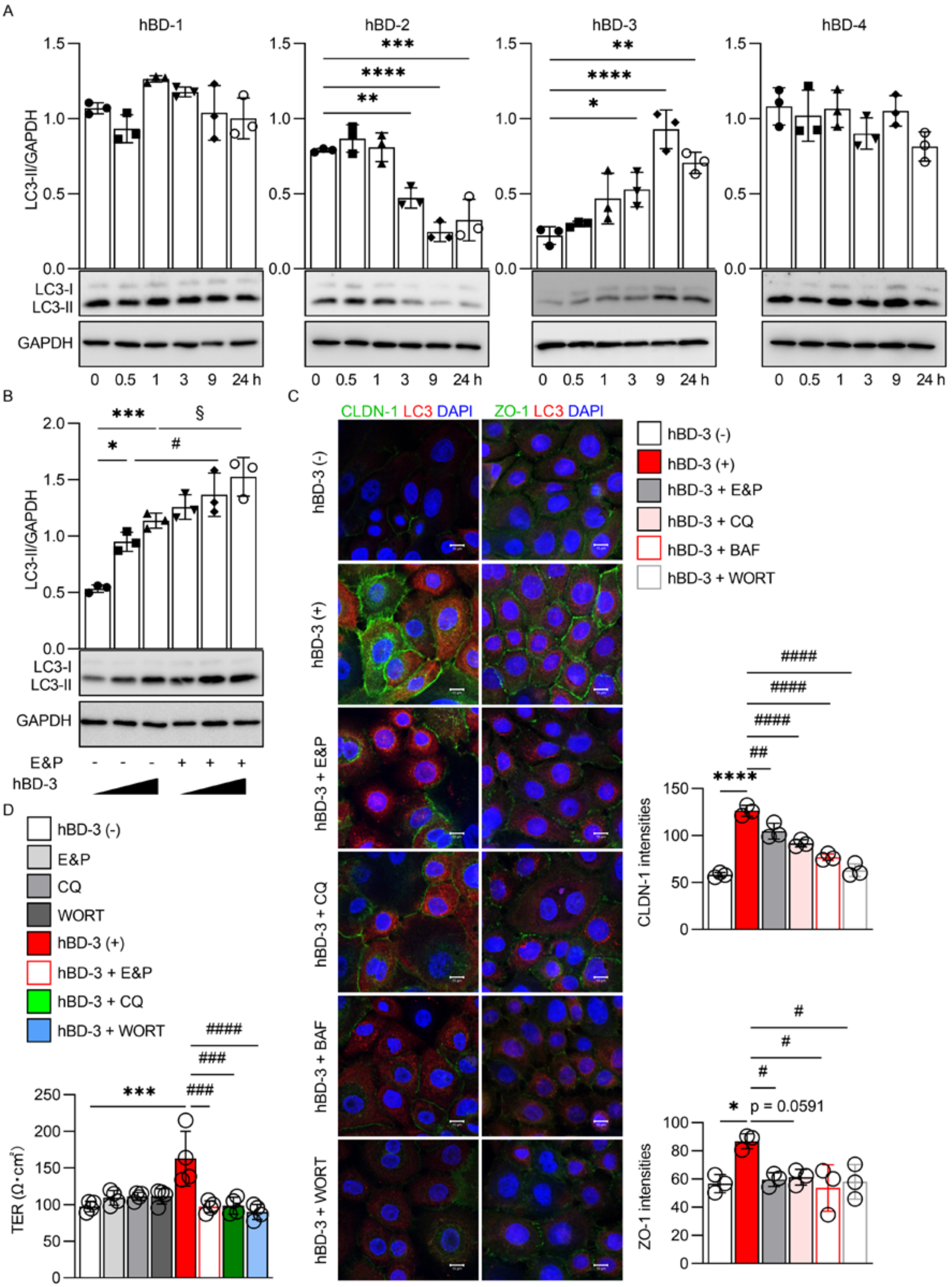

A
hBD-1
hBD-2
hBD-3
hBD-4
LC3-II/GAPDH
LC3-I
LC3-II
GAPDH
0 0.5 1 3 9 24 h
B
C
CLDN-1 LC3 DAPI
ZO-1 LC3 DAPI
hBD-3 (-)
hBD-3 (+)
hBD-3 + E&P
hBD-3 + CQ
hBD-3 + BAF
hBD-3 + WORT
E&P
hBD-3
D
E&P
CQ
WORT
hBD-3 + CQ
hBD-3 + WORT
CLDN-1 intensities
ZO-1 intensities
p = 0.0591
TER (Ω·cm²)

Figure S4. hBD-3 improves TJ barrier function in keratinocytes through autophagy activation

(A) Keratinocytes were treated with 10 μg/ml hBD-1, hBD-2, hBD-3, or hBD-4 for indicated time. Representative LC3 immunoblots (bottom) and quantification of band intensities (upper) are shown, n=3/group. GAPDH was used as a loading control. (B) Keratinocytes were pretreated with 10 μg/ml E&P or 0.1% DMSO for 2 hours and then treated with 10 μg/ml hBD-3 for 9 hours. Representative LC3 immunoblots (bottom) and quantification of band intensities (upper) are shown, n=3/group. (C) Keratinocytes were pretreated with 10 μg/ml E&P, 10 μM chloroquine (CQ), 10 μg/ml bafilomycin A1 (BAF), 10 μM wortmannin (WORT) or 0.1% DMSO for 2 hours and then treated with 10 μg/ml hBD-3 for 9 hours. Representative immunofluorescence images (right) and quantification of claudin-1 and ZO-1 (left) are shown, n = 3/group. Scale bar: 10 μm. (D) Keratinocyte layers grown on Transwell inserts were stimulated with E&P, chloroquine (CQ), wortmannin (WORT) or 0.1% DMSO alone or in combination with 10 μg/ml hBD-3 for 48 hours, and the transepithelial electrical resistance (TER) was assessed by CellZscope. $^{*}P < 0.05$, $^{**}P < 0.01$, $^{***}P < 0.001$, $^{****}P < 0.0001$, $^{\#}P < 0.05$, $^{\#\#}P < 0.01$, $^{\#\#\#}P < 0.001$, $^{\#\#\#\#}P < 0.0001$, $^{\S}P < 0.05$. Statistical significance was determined by one-way ANOVA with Tukey's multiple comparisons test. All of the data are representative of three independent experiments.

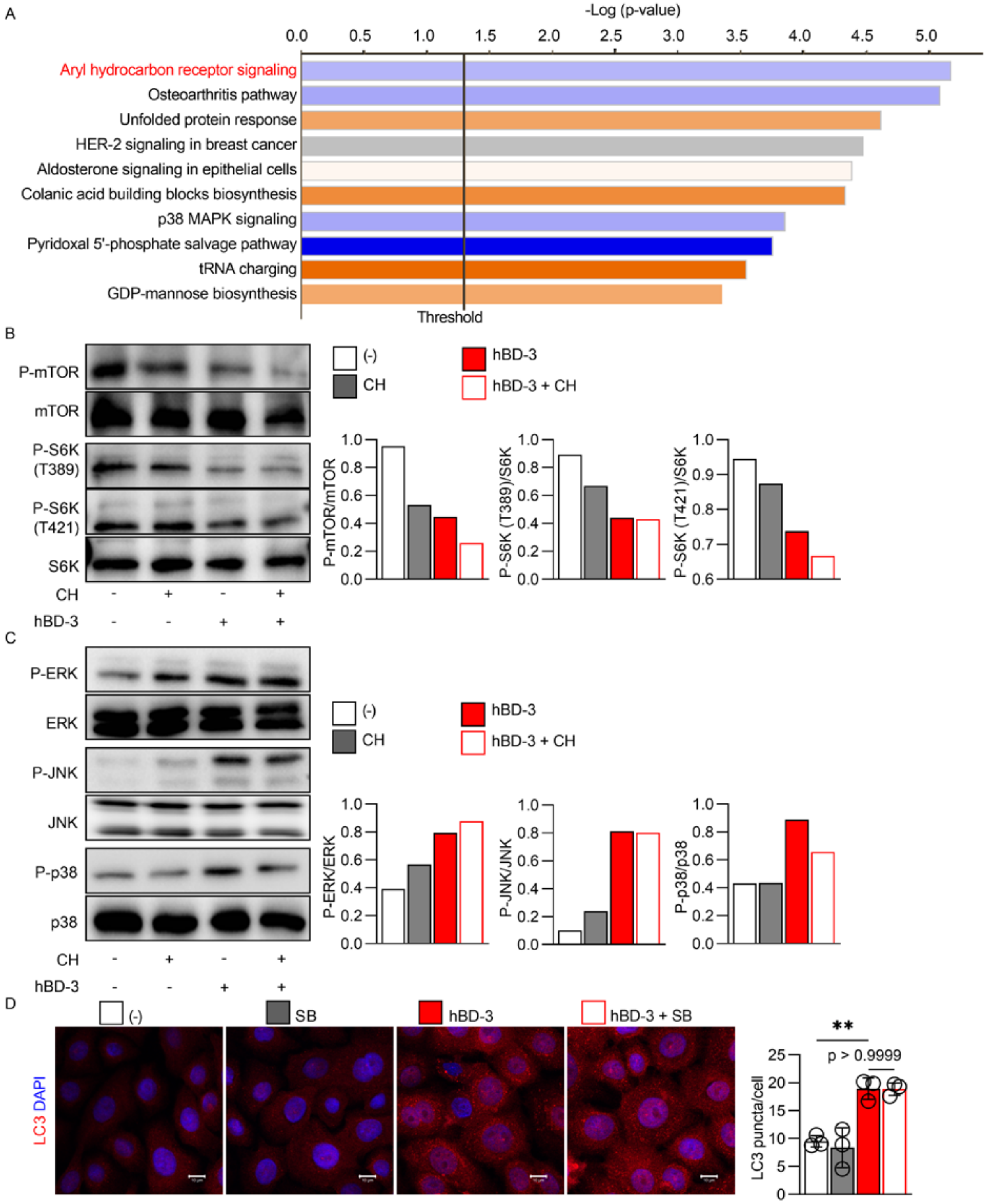

Figure S5. **hBD-3-induced autophagy is mTOR- and MAPK-independent**

(A) Ingenuity pathway analysis of DNA microarray data of autophagy-deficient keratinocytes and normal keratinocytes. (B) Keratinocytes were pretreated with CH-223191 (CH) for 2 hours and then treated with 10 µg/ml hBD-3 for 9 hours. GAPDH was used as a loading control. Representative immunoblots (left) and quantification of band intensities (right) are shown, n=3/group. (C) Keratinocytes were pretreated with CH-223191 (CH) for 2 hours and then treated with 10 µg/ml hBD-3 for 30 minutes. GAPDH was used as a loading control. Representative immunoblots (left) and quantification of band intensities (right) are shown, n=3/group. (D) Keratinocytes were pretreated with SB203580 (SB) for 2 hours and then treated with 10 µg/ml hBD-3 for 9 hours. Representative immunofluorescence images (left) and quantification of LC3 puncta (right) are shown, n = 3/group. Scale bar: 10 µm. ***P* < 0.01. Statistical significance was determined by one-way ANOVA with Tukey's multiple comparisons test.

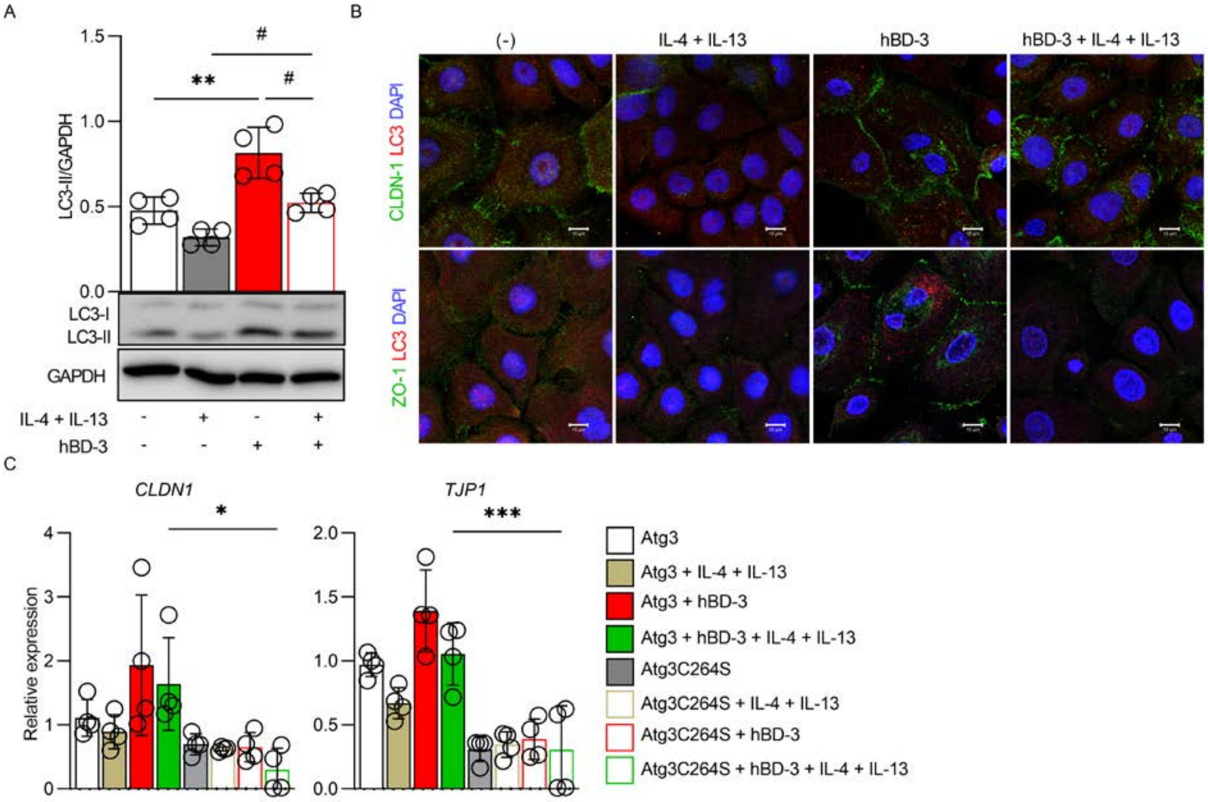

Figure S6. hBD-3 recovers IL-4- and IL-13-mediated disruption of TJ barrier through autophagy activation

(A-B) Keratinocytes were pretreated with 100 ng/ml IL-4 and 100 ng/ml IL-13 for 48 hours and then treated with 10 μg/ml hBD-3 for 9 hours. (A) Representative LC3 immunoblots (bottom) and quantification of band intensities (upper) are shown, n=3/group. GAPDH was used as a loading control. (B) Representative immunofluorescence images of claudin-1 and ZO-1 in keratinocytes, n = 4/group. Scale bar: 10 μm. (C) Keratinocytes were transfected with adenoviruses carrying mutant Atg3 or Atg3C264S for 48 hours and then treated with 100 ng/ml IL-4 and 100 ng/ml IL-13 for 24 hours and 10 μg/ml hBD-3 for 9 hours. Real-time PCR analysis of the indicated genes was performed. The data are presented as the mean ± SD. $^*P < 0.05$, $^{**}P < 0.01$, $^{***}P < 0.001$, $^{\#}P <$

0.05, ##$P < 0.01$. Statistical significance was determined by one-way ANOVA with Tukey's multiple comparisons test. All of the data are representative of three independent experiments.

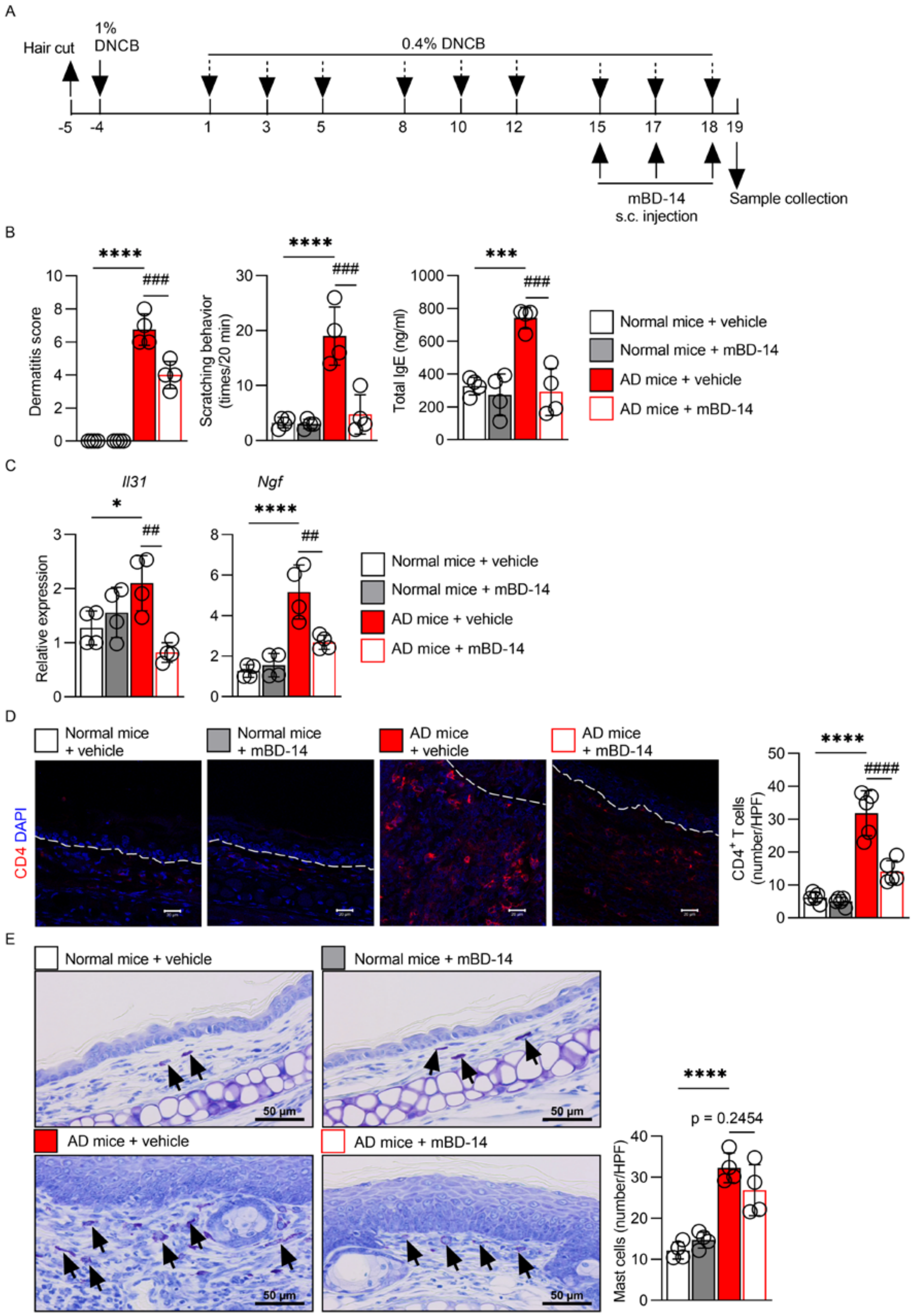
A
Hair cut
1%
DNCB
0.4% DNCB
-5
-4
1
3
5
8
10
12
15
17
18
19
mBD-14
s.c. injection
Sample collection
B
****
###
Dermatitis score
****
###
Scratching behavior
(times/20 min)
***
###
Total IgE (ng/ml)
Normal mice + vehicle
Normal mice + mBD-14
AD mice + vehicle
AD mice + mBD-14
C
Il31
*
##
Relative expression
Ngf
****
##
D
Normal mice
+ vehicle
Normal mice
+ mBD-14
AD mice
+ vehicle
AD mice
+ mBD-14
CD4 DAPI
CD4+ T cells
(number/HPF)
****
####
E
50 µm
Mast cells (number/HPF)
****
p = 0.2454

Figure S7. mBD-14 improves the symptoms of AD mice

(A) Scheme of the DNCB-induced AD mouse model and treatment with mBD-14. (B) Evaluation of the dermatitis score, scratching behavior and total serum IgE of mice on Day 19, n=4/group. (C) Real-time PCR analysis of the indicated genes in mouse ear samples, n=4/group. (D) Representative immunofluorescence images of $CD4^+$ T cells in the mouse epidermis (left) and quantification of the number of $CD4^+$ T cells in the ear skin (right), n=5/group. The white dashed line indicates the basement membrane between the epidermis and dermis. Scale bars: 10 μm. (E) Representative images of mast cells in the mouse ear identified using toluidine blue staining (left) and quantification of the number of mast cells in ear skin (right), n=5/group. Scale bars: 200 μm. $^{*}P < 0.05$, $^{***}P < 0.001$, $^{****}P < 0.0001$, $^{\#\#}P < 0.01$, $^{\#\#\#}P < 0.001$, $^{\#\#\#\#}P < 0.0001$. Statistical significance was determined by one-way ANOVA with Tukey's multiple comparisons test. All of the data are representative of three independent experiments.

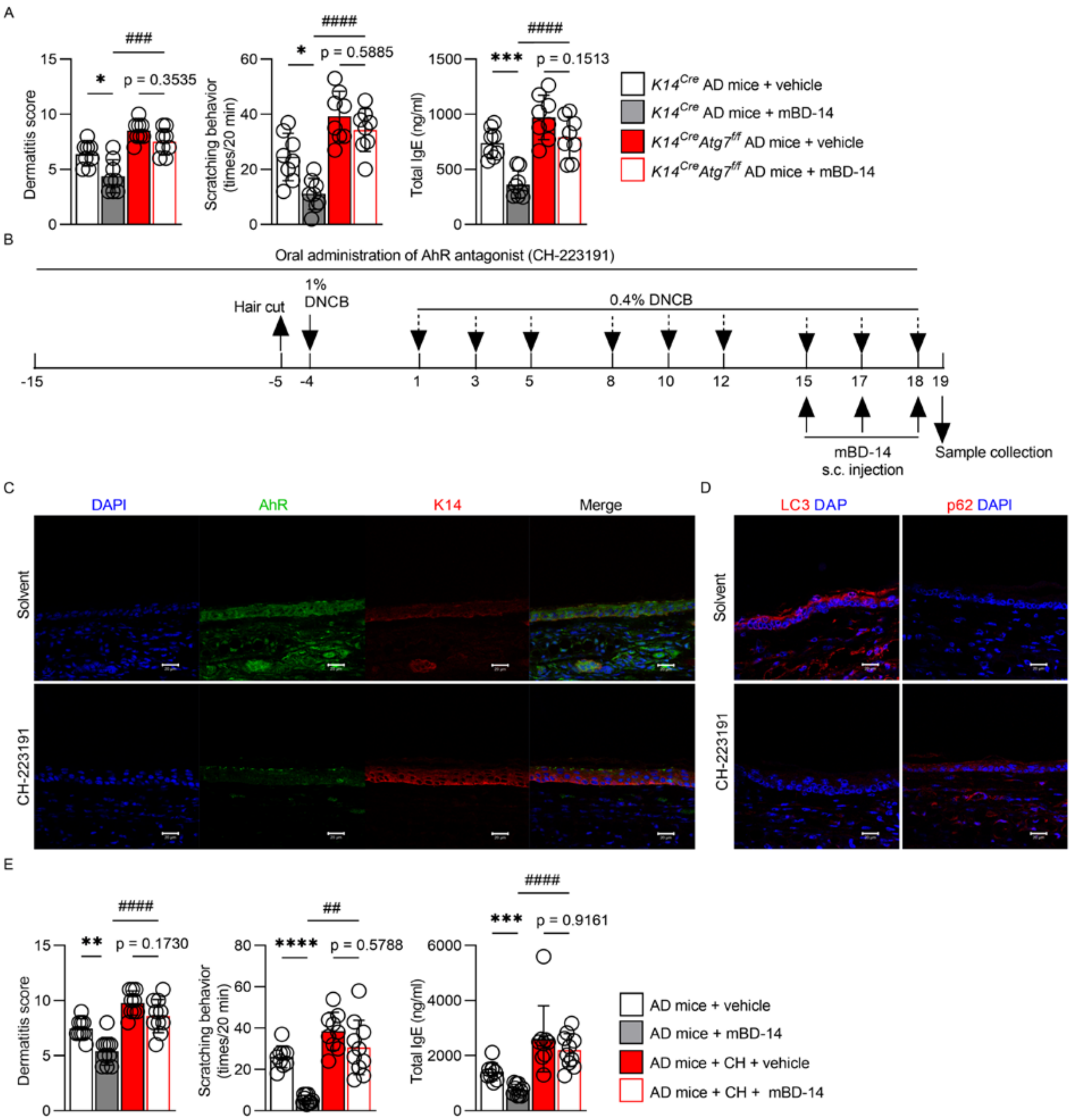

Figure S8. **Both autophagy activation and AhR signaling are required for mBD-14-mediated improvement in AD mice**

(A) Evaluation of the dermatitis score, scratching behavior and total serum IgE of mice on Day 19, n=8/group. (B) Scheme of the DNCB-induced AD mouse model using solvent-treated mice or CH-

2231991-treated mice. (C) Immunofluorescence staining of AhR and K14 in the epidermis of solvent-treated mice and CH-2231991-treated mice. (D) Immunofluorescence staining of LC3 and p62 in the epidermis of solvent-treated mice and CH-2231991-treated mice. Scale bar: 10 μm. (E) Evaluation of the dermatitis score, scratching behavior and total serum IgE of mice on Day 19, n=8/group. The data are presented as the mean ± SD. $^{*}P < 0.05$, $^{**}P < 0.01$, $^{***}P < 0.001$, $^{****}P < 0.0001$, $^{\#\#}P < 0.01$, $^{\#\#\#}P < 0.001$, $^{\#\#\#\#}P < 0.0001$. Statistical significance was determined by one-tailed Student's *t* test. All of the data are representative of three independent experiments.

Table S1. Genotyping primer sequences used for PCR

Primer name	Primer sequence (5'- to -3')
**Genotyping of *Atg7 flox***	
Transgene F	TGGCTGCTACTTCTGCAATGATGT
Transgene R	CAGGACAGAGACCATCAGCTCCAC
**Genotyping of *K14-Cre***	
Transgene F	TTCCTCAGGAGTGTCTTCGC
Transgene R	GTCCATGTCCTTCCTGAAGC

Table S2. List of antibodies used in this study

Primary antibodies			
**Antibodies**	**Catalog No.**	**Dilution**	**Company**
LC3	PM036	1:1000/1:2000	MBL, Nagoya, Japan
p62	5114S	1:1000/1:300	Cell Signaling Technology, Beverly, MA
Claudin-1	ab15098	1:300	Abcam, Waltham, MA
Claudin-1	374900	1:100	Invitrogen, Waltham, MA
ZO-1	339100	1:100	Invitrogen, Waltham, MA
Filaggrin	ab24584	1:1000	Abcam, Waltham, MA
Loricrin	ab85679	1:1000	Abcam, Waltham, MA
Involucrin	924401	1:500	Biolegend, San Diego, CA
AhR	17840-1-AP	1:1000	Proteintech, Sankt Leon-Rot, Germany
CYP1A1	sc-393979	1:1000	Santa Cruz Biotechnology, Dallas, TX
GAPDH	60004-1-lg	1:50000	Proteintech, Sankt Leon-Rot, Germany
AhR (A-3)	sc-133088	1:300	Santa Cruz Biotechnology, Dallas, TX

Anti-multi ubiquitin mAb	D058-3	1:1000	MBL, Nagoya, Japan
P-mTOR	2971S	1:2000	Cell Signaling Technology, Beverly, MA
mTOR	2972S	1:2000	Cell Signaling Technology, Beverly, MA
P-S6K (T389)	97596S	1:2000	Cell Signaling Technology, Beverly, MA
P-S6K (T421)	9204S	1:2000	Cell Signaling Technology, Beverly, MA
S6K	2708S	1:2000	Cell Signaling Technology, Beverly, MA
P-ERK	9101S	1:2000	Cell Signaling Technology, Beverly, MA
ERK	9102S	1:2000	Cell Signaling Technology, Beverly, MA
P-JNK	9251S	1:2000	Cell Signaling Technology, Beverly, MA
JNK	9252S	1:2000	Cell Signaling Technology, Beverly, MA
P-p38	9211S	1:2000	Cell Signaling Technology, Beverly, MA
p38	9212S	1:2000	Cell Signaling Technology, Beverly, MA
K14	GP-CK14	1:500	PROGEN, Heidelberg, Germany

Atg7	8558S	1:2000	Cell Signaling Technology, Beverly, MA
CD4	553049	1:100	BD Biosciences, Franklin Lakes, NJ
**Secondary antibodies**			
Alexa Fluor 594 goat anti-rabbit IgG (H+L)	A11037	1:1000	Invitrogen, Waltham, MA
Alexa Fluor 488 goat anti-rabbit IgG (H+L)	A11034	1:1000	Invitrogen, Waltham, MA
Alexa Fluor 594 goat anti-mouse IgG (H+L)	A11032	1:1000	Invitrogen, Waltham, MA
Alexa Fluor 594 goat anti-guinea pig IgG (H+L)	A-11076	1:1000	Invitrogen, Waltham, MA
Alexa Fluor 488 goat anti-mouse IgG (H+L)	A11029	1:1000	Invitrogen, Waltham, MA
Streptavidin Alexa Fluor 594-conjugated antibody	S11227	1:1000	Invitrogen, Waltham, MA
Sheep anti-rabbit antibody conjugated to HRP	NA934V	1:5000	Cytiva, Marlborough, MA
Sheep anti-mouse antibody conjugated to HRP	NA931V	1:5000	Cytiva, Marlborough, MA

Table S3. Primer sequences used for real-time PCR

Primer name	Primer sequence (5'- to -3')
hRPS18	
F	TTTGCGAGTACTCAACACCAACATC
R	GAGCATATCTTCGGCCCACAC
CLDN1	
F	GGGCAGATCCAGTGCAAAG
R	GGATGCCAACCACCATCAAG
TJP1	
F	GACCAATAGCTGATGTTGCCAGAG
R	TGCAGGCGAATAATGCCAGA
FLG	
F	GGAATTTCGGCAAATCCTG
R	GCTTGAGCCAACTTGAATACCA
LOR	
F	GGCTGCATCTAGTTCTGCTGTTTA
R	CAAATTTATTGACTGAGGCACTGG
Actb	
F	CATCCGTAAAGACCTCTCTATGCCAAC
R	ATGGAGCCACCGATCCACA
Cldn1	
F	ACCGGGCAGATACAGTGCAA
R	TGCCAATGGTGGACACAAAGA
Tjp1	
F	GTTGGTACGGTGCCCTGAAAGA
R	GCTGACAGGTAGGACAGACGAT

Il4	
F	GACTCGCCTACAAAGCCCAGA
R	AGCTGCTTGTGCCTGTGGAA
Il13	
F	CCCTGGAATCCCTGATCAAC
R	CCTTTACAAACTGGGCCACCTC
Il33	
F	GTTGCATGCCAACAACAAGGA
R	GCATTCAAATGAAACACAGTTGGAG
Tslp	
F	CGCCTATGAGCAGCCACATT
R	TCTTCTTCATTGCCTGAGTAGCATT

Table S4. Known LIR-containing proteins, their LIR motifs and potential AhR LIR motifs

LIR proteins (UniProt entry No.)	LIR motif	LIR position	Ref
NDP52 (Q13137)	ENEEDI**LVV**TT	134	(1, 2)
Tax1p1/T6BP (Q86VP1)	EGNSDM**LVV**TT	141	(3)
ULK2 (Q8IYT8)	SCDTDD**FVLV**PH	353	(4)
DmATG1B (Q8MQJ7)	HEDSDD**FVLV**PK	391	(4)
AhR (P35869)	ALNGFV**LVV**T	127	NA
	ALNG**FVLV**VT	125	NA

Bolded amino acids indicate the core consensus sequence.

References

1. Thurston TL, Ryzhakov G, Bloor S, von Muhlinen N, and Randow F. The TBK1 adaptor and autophagy receptor NDP52 restricts the proliferation of ubiquitin-coated bacteria. *Nat Immunol.* 2009;10(11):1215-21.

2. von Muhlinen N, Akutsu M, Ravenhill BJ, Foeglein Á, Bloor S, Rutherford TJ, et al. LC3C, bound selectively by a noncanonical LIR motif in NDP52, is required for antibacterial autophagy. *Mol Cell.* 2012;48(3):329-42.

3. Newman AC, Scholefield CL, Kemp AJ, Newman M, McIver EG, Kamal A, et al. TBK1 kinase addiction in lung cancer cells is mediated via autophagy of Tax1bp1/Ndp52 and non-canonical NF-κB signalling. *PLoS One.* 2012;7(11):e50672.

4. Alemu EA, Lamark T, Torgersen KM, Birgisdottir AB, Larsen KB, Jain A, et al. ATG8 family proteins act as scaffolds for assembly of the ULK complex: sequence requirements for LC3-interacting region (LIR) motifs. *J Biol Chem.* 2012;287(47):39275-90.
